# Clinical characteristics and outcomes for 7,995 patients with SARS-CoV-2 infection

**DOI:** 10.1371/journal.pone.0243291

**Published:** 2021-03-31

**Authors:** Jacob McPadden, Frederick Warner, H. Patrick Young, Nathan C. Hurley, Rebecca A. Pulk, Avinainder Singh, Thomas J. S. Durant, Guannan Gong, Nihar Desai, Adrian Haimovich, Richard Andrew Taylor, Murat Gunel, Charles S. Dela Cruz, Shelli F. Farhadian, Jonathan Siner, Merceditas Villanueva, Keith Churchwell, Allen Hsiao, Charles J. Torre, Eric J. Velazquez, Roy S. Herbst, Akiko Iwasaki, Albert I. Ko, Bobak J. Mortazavi, Harlan M. Krumholz, Wade L. Schulz

**Affiliations:** 1 Department of Pediatrics, Yale School of Medicine, New Haven, Connecticut, United States of America; 2 Center for Outcomes Research and Evaluation, Yale-New Haven Hospital, New Haven, Connecticut, United States of America; 3 Department of Internal Medicine, Section of Cardiovascular Medicine, Yale School of Medicine, New Haven, Connecticut, United States of America; 4 Department of Internal Medicine, Yale University School of Medicine, New Haven, Connecticut, United States of America; 5 Department of Computer Science and Engineering, Texas A&M University, College Station, Texas, United States of America; 6 Corporate Pharmacy Services, Yale New Haven Health, New Haven, Connecticut, United States of America; 7 Department of Laboratory Medicine, Yale University School of Medicine, New Haven, Connecticut, United States of America; 8 Interdepartmental Program in Computational Biology and Bioinformatics, Yale University School of Medicine, New Haven, Connecticut, United States of America; 9 Yale School of Medicine, New Haven, Connecticut, United States of America; 10 Department of Emergency Medicine, Yale School of Medicine, New Haven, Connecticut, United States of America; 11 Department of Genetics, Yale University School of Medicine, New Haven, Connecticut, United States of America; 12 Medical Scientist Training Program, Yale University School of Medicine, New Haven, Connecticut, United States of America; 13 Yale Center for Genome Analysis, Yale University School of Medicine, New Haven, Connecticut, United States of America; 14 Department of Neurosurgery, Yale University School of Medicine, New Haven, Connecticut, United States of America; 15 Department of Internal Medicine, Pulmonary, Critical Care and Sleep Medicine, Yale School of Medicine, New Haven, Connecticut, United States of America; 16 Department of Internal Medicine, Section of Infectious Diseases, Yale School of Medicine, New Haven, Connecticut, United States of America; 17 Center for Interdisciplinary Research on AIDS, Yale School of Public Health, New Haven, Connecticut, United States of America; 18 Yale New Haven Hospital, New Haven, Connecticut, United States of America; 19 Information Technology Services, Yale New Haven Health, New Haven, Connecticut, United States of America; 20 Yale Comprehensive Cancer Center, Yale School of Medicine, New Haven, Connecticut, United States of America; 21 Department of Immunobiology, Yale University School of Medicine, New Haven, Connecticut, United States of America; 22 Howard Hughes Medical Institute, Chevy Chase, Maryland, United States of America; 23 Department of Epidemiology of Microbial Diseases, Yale School of Public Health, New Haven, Connecticut, United States of America; 24 Center for Remote Health Technologies and Systems, Texas A&M University, College Station, Texas, United States of America; 25 Department of Health Policy and Management, Yale School of Public Health, New Haven, Connecticut, United States of America; National Institute for Infectious Diseases Lazzaro Spallanzani-IRCCS, ITALY

## Abstract

**Objective:**

Severe acute respiratory syndrome virus (SARS-CoV-2) has infected millions of people worldwide. Our goal was to identify risk factors associated with admission and disease severity in patients with SARS-CoV-2.

**Design:**

This was an observational, retrospective study based on real-world data for 7,995 patients with SARS-CoV-2 from a clinical data repository.

**Setting:**

Yale New Haven Health (YNHH) is a five-hospital academic health system serving a diverse patient population with community and teaching facilities in both urban and suburban areas.

**Populations:**

The study included adult patients who had SARS-CoV-2 testing at YNHH between March 1 and April 30, 2020.

**Main outcome and performance measures:**

Primary outcomes were admission and in-hospital mortality for patients with SARS-CoV-2 infection as determined by RT-PCR testing. We also assessed features associated with the need for respiratory support.

**Results:**

Of the 28605 patients tested for SARS-CoV-2, 7995 patients (27.9%) had an infection (median age 52.3 years) and 2154 (26.9%) of these had an associated admission (median age 66.2 years). Of admitted patients, 2152 (99.9%) had a discharge disposition at the end of the study period. Of these, 329 (15.3%) required invasive mechanical ventilation and 305 (14.2%) expired. Increased age and male sex were positively associated with admission and in-hospital mortality (median age 80.7 years), while comorbidities had a much weaker association with the risk of admission or mortality. Black race (OR 1.43, 95%CI 1.14–1.78) and Hispanic ethnicity (OR 1.81, 95%CI 1.50–2.18) were identified as risk factors for admission, but, among discharged patients, age-adjusted in-hospital mortality was not significantly different among racial and ethnic groups.

**Conclusions:**

This observational study identified, among people testing positive for SARS-CoV-2 infection, older age and male sex as the most strongly associated risks for admission and in-hospital mortality in patients with SARS-CoV-2 infection. While minority racial and ethnic groups had increased burden of disease and risk of admission, age-adjusted in-hospital mortality for discharged patients was not significantly different among racial and ethnic groups. Ongoing studies will be needed to continue to evaluate these risks, particularly in the setting of evolving treatment guidelines.

## Introduction

Severe acute respiratory syndrome virus (SARS-CoV-2) has infected over 110 million people with nearly 2.5 million deaths worldwide [1]. Despite the global impact, key gaps in knowledge persist. A comprehensive assessment of patients evaluated for SARS-CoV-2, from testing to outcome, is needed to guide public health recommendations and scientific investigations into the mechanisms of disease pathogenesis.

Prior studies have identified many risk factors for SARS-CoV-2 infections and complications [2–5]. Older age and male sex have been consistently associated with worse outcomes, as have many chronic cardiovascular and respiratory diseases [3–6]. Despite some consistent themes, reports from different geographic locations have reported variation in both risks and mortality rates [7–11]. No study yet exists that describes the characteristics and outcomes of a single cohort from testing to outcome and with detailed information on treatments in a racially and ethnically diverse population.

Drawing from a highly curated real-world data set, we describe a diverse cohort from a catchment area that represents the diversity of the nation located in an early epicenter of the US outbreak. We extend the current literature with a detailed assessment of the characteristics of patients tested, and the clinical courses and outcomes of those testing positive, and among those admitted with SARS-CoV-2. We sought to identify risk factors for admission among those with SARS-CoV-2 and in-hospital mortality among discharged patients. We also characterize the patterns of treatment to provide the context to guide interpretation of these results.

## Methods

### Study setting and data collection

This was an observational, retrospective study of patients who were tested for SARS-CoV-2 within the Yale New Haven Health (YNHH) system, located within one of the US epicenters of Covid-19. The healthcare system is comprised of a mix of pediatric, suburban community, urban community, and urban academic inpatient facilities at five sites with a total of 2,681 licensed beds and 124,668 inpatient discharges in 2018 [12]. The system also includes associated outpatient facilities that had 2.4 million outpatient encounters in 2018. YNHH uses a single electronic health record (EHR) across the health system. Patient demographics, past medical histories, medications, and clinical outcomes were extracted from our local Observational Medical Outcomes Partnership (OMOP) [13] data repository and analyzed within our computational health platform [14, 15]. Data were extracted with custom PySpark (version 2.4.5) scripts that were reviewed by an independent analyst. The study was approved by the Yale University Institutional Review Board (protocol #2000027747).

### Study cohort

The study cohort consists of all adult patients (≥18 years old) at YNHH who had an order for SARS-CoV-2 RT-PCR testing and a test result documented within the medical record between March 1, 2020 and April 30, 2020 (S1 Fig). SARS-CoV-2 testing in our health system was limited to symptomatic patients for whom the provider had a concern for respiratory tract infection in the month of March. Testing increased to include a wider breadth of symptoms deemed clinically concerning during the month of April. By the end of April, all patients admitted to the health system were tested for Covid-19. Outpatient testing required a physician order and was primarily sent to external reference laboratories. The decision to test was ultimately left to the ordering provider. Testing was first made available to order within the health system on March 13th, 2020.

Patients admitted more than 24 hours prior to testing were excluded from the admissions group to reduce the likelihood of including hospital acquired infections. Data and outcomes were limited to those collected between March 1, 2020 and April 30, 2020. An extract of our local OMOP data repository from September 13, 2020 was used to allow for final discharge disposition and vendor-provided transformations of the clinical data warehouse to complete. For patients with multiple admissions in the study period, only data from the first admission was used. Race and ethnicity were extracted from the demographics section of the EHR and mapped to the OMOP common data model (S1 Table). For demographic fields that had selected values or responses, individual counts were further anonymized to remove any counts ≤3.

### Outcome ascertainment

We extracted primary outcomes of admission and discharge disposition along with secondary outcomes of supplemental oxygen use and mechanical respiratory support. The maximum respiratory requirement during admission was used. Covid-19 related admissions were identified by extracting data from each patient’s first inpatient admission that had a visit start time within a window 14 days following or 24 hours before a positive SARS-CoV-2 test was ordered for a patient. For patients with a transfer to another facility (n = 44), the outcome from the first visit was used. Visit-related data and in-hospital mortality were directly extracted from our OMOP data repository. Supplemental oxygen requirements were computed based on presence of clinical documentation in flowsheets or vitals measurements and were mapped to one of four categorical variables: low-flow oxygen, high-flow oxygen, noninvasive mechanical ventilation, and/or invasive mechanical ventilation. Outcomes were limited to patients who were discharged and were therefore not extracted for patients who were still admitted at the end of the study period. Digitally extracted outcomes were validated for 30 patients via medical record review by a clinician. All ages were calculated relative to the time of SARS-CoV-2 test order.

### Treatment pathways

To document clinical treatment pathways, we extracted medication administration records of all admitted patients for their initial visit. Medications related to Covid-19 treatment based on institutional guidelines were grouped by calendar day of first administration. All forms of corticosteroids were mapped to a single drug class rather than their individual active ingredients. The order of medication initiation defined the separate treatment regimens and final treatment pathway. Treatment pathway visualizations were created with the JavaScript library Data Driven Documents (D3, version 4) [16].

### Statistical analyses

The tables of demographic data and outcome data were built using the R (version 3.5.1) package tableone. Logistic regressions were performed using the core R function glm. Model 1 was among those testing positive to identify risk factors associated with admission. Candidate variables included the features described in Table 1. Before computing the final model, the variables for "Other" race and ethnicity of "Not Hispanic" were removed in order to ensure that all variables in the model had variance inflation factor less than 3. Model 2 was among those with a final discharge disposition at the end of the study period (right-censored for patients who were still admitted) to identify risk factors associated with in-hospital mortality. We began with the variables used in the admission model and removed the race variables for “American Indian or Alaska Native” and “Native Hawaiian or Other Pacific Islander” and the age variable “Age 35–44”. This was done to ensure the variation inflation factors would all be less than 10. A value of p<0.05 was used as the threshold for significance without adjustment for multiple comparisons.

**Table 1:** Demographics and Elixhauser comorbidities of all patients tested, tested positive, and admitted for SARS-CoV-2.

	Tested	Positive	Admitted
n	28605	7995	2154
Sex (%)			
Female	17191 (60.1)	4435 (55.5)	1031 (47.9)
Male	11404 (39.9)	3558 (44.5)	1123 (52.1)
Unknown	10 (0.0)	2 (0.0)	0 (0.0)
Race (%)			
American Indian	60 (0.2)	12 (0.2)	<10 (<0.5)
Asian	721 (2.5)	175 (2.2)	44 (2.0)
Black	5093 (17.8)	1856 (23.2)	546 (25.3)
Hawaiian/Pacific Islander	79 (0.3)	30 (0.4)	<10 (<0.5)
Other	4464 (15.6)	1874 (23.4)	497 (23.1)
Unknown/Not Stated	1363 (4.8)	432 (5.4)	37 (1.7)
White	16825 (58.8)	3616 (45.2)	1021 (47.4)
Ethnicity (%)			
Hispanic or Latino	5468 (19.1)	2245 (28.1)	560 (26.0)
Not Hispanic or Latino	21540 (75.3)	5265 (65.9)	1559 (72.4)
Unknown/Not Stated	1597 (5.6)	485 (6.1)	35 (1.6)
Age (%)			
18–34	6581 (23.0)	1579 (19.7)	146 (6.8)
35–44	4880 (17.1)	1321 (16.5)	181 (8.4)
45–54	5207 (18.2)	1546 (19.3)	258 (12.0)
55–64	5480 (19.2)	1583 (19.8)	435 (20.2)
65–74	3194 (11.2)	885 (11.1)	402 (18.7)
75–84	1928 (6.7)	565 (7.1)	374 (17.4)
85+	1335 (4.7)	516 (6.5)	358 (16.6)
Elixhauser Comorbidities (%)			
AIDS/HIV	248 (0.9)	75 (0.9)	32 (1.5)
Alcohol abuse	2141 (7.5)	398 (5.0)	175 (8.1)
Blood loss anemia	1228 (4.3)	305 (3.8)	144 (6.7)
Cardiac arrhythmias	7242 (25.3)	1691 (21.2)	803 (37.3)
Chronic pulmonary disease	9043 (31.6)	2005 (25.1)	717 (33.3)
Coagulopathy	2450 (8.6)	511 (6.4)	265 (12.3)
Congestive heart failure	3155 (11.0)	805 (10.1)	494 (22.9)
Deficiency anemia	3787 (13.2)	978 (12.2)	409 (19.0)
Depression	7697 (26.9)	1664 (20.8)	637 (29.6)
Diabetes, complicated	3817 (13.3)	1190 (14.9)	624 (29.0)
Diabetes, uncomplicated	5502 (19.2)	1738 (21.7)	810 (37.6)
Drug abuse	2548 (8.9)	401 (5.0)	173 (8.0)
Fluid and electrolyte disorders	6540 (22.9)	1623 (20.3)	905 (42.0)
Hypertension, complicated	3666 (12.8)	988 (12.4)	616 (28.6)
Hypertension, uncomplicated	11950 (41.8)	3334 (41.7)	1387 (64.4)
Hypothyroidism	4467 (15.6)	1154 (14.4)	448 (20.8)
Liver disease	3417 (11.9)	755 (9.4)	283 (13.1)
Lymphoma	421 (1.5)	71 (0.9)	29 (1.3)
Metastatic cancer	1557 (5.4)	271 (3.4)	140 (6.5)
Obesity	7654 (26.8)	2195 (27.5)	684 (31.8)
Other neurological disorders	3481 (12.2)	939 (11.7)	542 (25.2)
Paralysis	672 (2.3)	203 (2.5)	118 (5.5)
Peptic ulcer disease, excluding bleeding	989 (3.5)	217 (2.7)	113 (5.2)
Peripheral vascular disorders	3416 (11.9)	875 (10.9)	523 (24.3)
Psychoses	1259 (4.4)	330 (4.1)	206 (9.6)
Pulmonary circulation disorders	1568 (5.5)	358 (4.5)	226 (10.5)
Renal failure	2888 (10.1)	809 (10.1)	515 (23.9)
Rheumatoid arthritis/collagen vascular diseases	2145 (7.5)	432 (5.4)	174 (8.1)
Solid tumor without metastasis	3023 (10.6)	658 (8.2)	313 (14.5)
Valvular disease	4188 (14.6)	1011 (12.6)	528 (24.5)
Weight loss	2864 (10.0)	664 (8.3)	357 (16.6)

Elixhauser comorbidity [17] analysis was performed using the R comorbidity package (version 0.5.3) [18]. Briefly, ICD-10 codes from each patient’s medical history taken from the OMOP database were used to generate presence or absence of the 31 Elixhauser comorbidity categories, as well as weighted scores using the AHRQ and van Walraven algorithms [19, 20].

Age-adjusted in-hospital mortality was calculated with direct standardization [21] based on the discharge population. In this method, age-specific rates are weighted according to the prevalence of age groups within an a priori standard population. This converts the observed age-specific rates of some process into a rate which would be observed had that same process acted upon the standard population. The 2000 US population was used as the standard population for age adjustment [21]. We used weights for five-year age groupings from ages 15 to 84 and a final group of 85 and over.

## Results

The number of patients positive for SARS-CoV-2 increased rapidly beginning in March 2020 (S2 Fig). A total of 28605 patients were tested for SARS-CoV-2 with 7995 patients (27.9%) who had at least one positive result during the observation period. Of those with positive tests, 2154 (26.9%) had an associated hospital admission. Of admitted patients, 2152 (99.9%) had a final discharge disposition and 2 (0.1%) remained hospitalized at the time of data extraction. For SARS-CoV-2 infected patients who were not admitted, the median number of days elapsed between testing and the study end date was 23.4 days (IQR 14.6–30.6).

### Characteristics of individuals tested for SARS-CoV-2

Of the patients tested for SARS-CoV-2, a majority (n = 17191; 60.1%) were female (Table 1). The most common comorbidities were uncomplicated hypertension (n = 11950; 41.8%), chronic pulmonary disease (n = 9043; 31.6%), and depression (n = 7697; 26.9%). The median age of tested adults was 50.8 years (IQR 36.1–63.5). In those tested for SARS-CoV-2, 4.8% did not have a reported race within the demographics section of the EHR. The majority of tested patients were reported as White (n = 16825; 58.8%), followed by Black (n = 5093; 17.8%) and Other race (n = 4464; 15.6%). Those who self-identified as Hispanic ethnicity represented 19.1% (n = 5468) of the tested population. Testing frequency by race and ethnicity showed slight overrepresentation of minority groups based on the census numbers for Connecticut, which has a demographic breakdown of 66.9% White, 12.2% Black, 5.0% Asian, 0.6% American Indian or Alaskan Native, 0.1% Native Hawaiian or Pacific Islander, and 16.9% Hispanic [22].

Age was similarly distributed between the SARS-CoV-2 tested and positive populations. Of those who tested positive, the median age was 52.3 years (IQR 38.3–64.8). Patients with a positive test were more frequently female (n = 4435, 55.5%) with uncomplicated hypertension (n = 3334, 41.7%), obesity (n = 2195, 27.5%), and chronic pulmonary disease (n = 2005, 25.1%) as the most common comorbidities. Patients with a positive test were most frequently reported as White (n = 3616, 45.2%), followed by Other race (n = 1874, 23.4%) and Black (n = 1856, 23.2%). Those who were reported as Hispanic ethnicity accounted for 28.1% (n = 2245) of SARS-CoV-2 positive patients.

### Features associated with admission in patients with Covid-19

The median age of SARS-CoV-2 positive patients admitted to the hospital was 66.2 years (IQR 53.7–79.9) and a majority were male (n = 1123, 52.1%) as shown in Table 1. The most common Elixhauser comorbidities for admitted patients included uncomplicated hypertension (n = 1387, 64.4%), fluid & electrolyte disorders (n = 905, 42.0%), and diabetes without complications (n = 810, 37.6%). Minority groups were overrepresented in the admitted population compared to census numbers, particularly for those with a recorded race of Black (n = 546, 25.3%) or Other race (n = 497, 23.1%). Those recorded as Hispanic ethnicity accounted for 26.0% (n = 560) of admitted patients.

**Fig 1:**
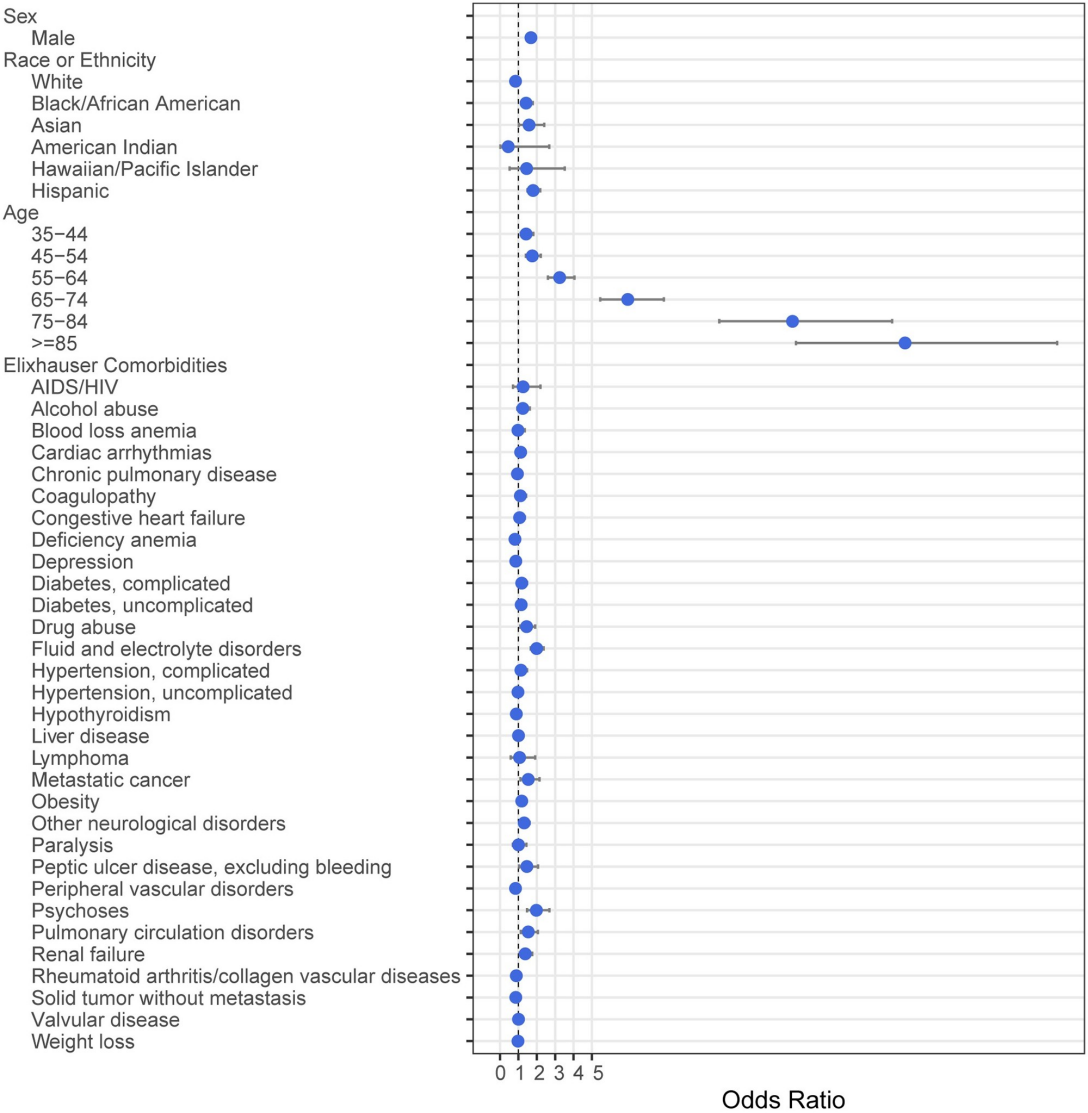
Multivariable analysis with odds ratios for admission in patients with a positive SARS-CoV-2 test. Bars indicate 95% confidence intervals.

In multivariable analyses, older age was significantly associated with risk of admission (Fig 1, S2 Table). Age ≥85 years had the highest risk of admission (OR 22.03, 95%CI = 16.10–30.30). Male sex was also associated with increased risk of admission (OR 1.68, 95%CI = 1.48–1.90). The comorbidities associated with increased risk of admission included fluid & electrolyte disorders (OR 1.99, 95%CI = 1.67–2.37), psychoses (OR 1.98, 95%CI = 1.47–2.69), metastatic cancer (OR 1.55, 95%CI = 1.11–2.15), pulmonary circulation disorders (OR 1.53, 95%CI = 1.14–2.06), peptic ulcer disease (OR 1.47, 95%CI = 1.04–2.07), drug abuse (OR 1.46, 95%CI = 1.11–1.92), renal failure (OR 1.38, 95%CI = 1.08–1.75), other neurological disorders (OR 1.31, 95%CI = 1.07–1.61), and obesity (OR 1.18, 95%CI = 1.02–1.37). Of note, complicated hypertension (OR 1.14, 95%CI = 0.88–1.48), uncomplicated hypertension (OR 0.97, 95%CI = 0.83–1.13), and chronic pulmonary disease (OR 0.94, 95%CI = 0.81–1.09) were not found to significantly increase the odds of admission. Recorded races with increased odds of admission included Asian (OR 1.58, 95%CI = 1.02–2.41) and Black (OR 1.43, 95%CI = 1.14–1.78). Hispanic ethnicity was also associated with increased risk of admission (OR 1.81, 95%CI = 1.50–2.18).

### Outcomes in discharged patients with Covid-19

Of the patients admitted for COVID-19 the majority (n = 2152, 99.9%) had a known disposition and therefore had complete outcomes available at the time of data extraction (Table 2). The median length of stay for discharged patients was 8.1 days (IQR 4.3–14.8). Mortality occurred in the emergency department for 8 of these patients who were excluded from respiratory analysis as they did not have complete respiratory outcomes reported.

**Table 2:** Discharge and respiratory outcomes (highest requirement during admission) for all patients with known disposition categorized by sex, race, ethnicity and Elixhauser comorbidities.

	Discharged	No Oxygen	Low Flow	High Flow	Noninvasive	Invasive	Discharged Alive	Expired
n	2152	430	809	511	73	329	1847	305
Sex								
Male (%)	1122 (52.1)	196 (45.6)	379 (46.8)	296 (57.9)	36 (49.3)	215 (65.3)	947 (51.3)	175 (57.4)
Race (%)								
American Indian or Alaska Native	<10 (<0.5)	0 (0.0)	<5 (<0.6)	0 (0.0)	0 (0.0)	0 (0.0)	<10 (<0.5)	0 (0.0)
Asian	44 (2.0)	8 (1.9)	13 (1.6)	13 (2.5)	<5 (<6.8)	<10 (<3.0)	38 (2.1)	<10 (<3.3)
Black	544 (25.3)	110 (25.6)	209 (25.8)	111 (21.7)	14 (19.2)	100 (30.4)	475 (25.7)	69 (22.6)
Hawaiian/Pacific Islander	<10 (<0.5)	0 (0.0)	<5 (<0.6)	<5 (<1.0)	0 (0.0)	<10 (<3.0)	<10 (<0.5)	<10 (<3.3)
Other	497 (23.1)	107 (24.9)	166 (20.5)	110 (21.5)	15 (20.5)	99 (30.1)	454 (24.6)	43 (14.1)
Unknown/Not Stated	37 (1.7)	7 (1.6)	19 (2.3)	<5 (<1.0)	<5 (<6.8))	<10 (<3.0)	37 (2.0)	0 (0.0)
White	1021 (47.4)	198 (46.0)	397 (49.1)	272 (53.2)	41 (56.2)	113 (34.3)	836 (45.3)	185 (60.7)
Ethnicity (%)								
Hispanic or Latino	560 (26.0)	125 (29.1)	196 (24.2)	111 (21.7)	18 (24.7)	110 (33.4)	511 (27.7)	49 (16.1)
Not Hispanic or Latino	1557 (72.4)	302 (70.2)	596 (73.7)	393 (76.9)	55 (75.3)	211 (64.1)	1308 (70.8)	249 (81.6)
Unknown/Not Stated	35 (1.6)	3 (0.7)	17 (2.1)	7 (1.4)	0 (0.0)	8 (2.4)	28 (1.5)	7 (2.3)
Age (%)								
18–34	146 (6.8)	73 (17.0)	35 (4.3)	18 (3.5)	4 (5.5)	16 (4.9)	145 (7.9)	1 (0.3)
35–44	181 (8.4)	61 (14.2)	58 (7.2)	28 (5.5)	4 (5.5)	30 (9.1)	176 (9.5)	5 (1.6)
45–54	257 (11.9)	54 (12.6)	104 (12.9)	48 (9.4)	8 (11.0)	43 (13.1)	248 (13.4)	9 (3.0)
55–64	435 (20.2)	67 (15.6)	166 (20.5)	101 (19.8)	14 (19.2)	87 (26.4)	399 (21.6)	36 (11.8)
65–74	402 (18.7)	60 (14.0)	171 (21.1)	81 (15.9)	17 (23.3)	73 (22.2)	351 (19.0)	51 (16.7)
75–84	373 (17.3)	58 (13.5)	137 (16.9)	104 (20.4)	18 (24.7)	56 (17.0)	278 (15.1)	95 (31.1)
85+	358 (16.6)	57 (13.3)	138 (17.1)	131 (25.6)	8 (11.0)	24 (7.3)	250 (13.5)	108 (35.4)
Comorbidity (%)								
AIDS/HIV	32 (1.5)	3 (0.7)	13 (1.6)	8 (1.6)	3 (4.1)	5 (1.5)	28 (1.5)	4 (1.3)
Alcohol abuse	175 (8.1)	35 (8.1)	68 (8.4)	39 (7.6)	6 (8.2)	27 (8.2)	151 (8.2)	24 (7.9)
Blood loss anemia	142 (6.6)	16 (3.7)	62 (7.7)	37 (7.2)	5 (6.8)	22 (6.7)	98 (5.3)	44 (14.4)
Cardiac arrhythmias	801 (37.2)	134 (31.2)	318 (39.3)	215 (42.1)	27 (37.0)	107 (32.5)	639 (34.6)	162 (53.1)
Chronic pulmonary disease	716 (33.3)	110 (25.6)	281 (34.7)	195 (38.2)	32 (43.8)	98 (29.8)	587 (31.8)	129 (42.3)
Coagulopathy	264 (12.3)	39 (9.1)	109 (13.5)	64 (12.5)	7 (9.6)	45 (13.7)	201 (10.9)	63 (20.7)
Congestive heart failure	493 (22.9)	61 (14.2)	197 (24.4)	145 (28.4)	19 (26.0)	71 (21.6)	380 (20.6)	113 (37.0)
Deficiency anemia	407 (18.9)	65 (15.1)	162 (20.0)	106 (20.7)	19 (26.0)	55 (16.7)	323 (17.5)	84 (27.5)
Depression	636 (29.6)	101 (23.5)	253 (31.3)	182 (35.6)	27 (37.0)	73 (22.2)	526 (28.5)	110 (36.1)
Diabetes, complicated	623 (28.9)	87 (20.2)	232 (28.7)	159 (31.1)	27 (37.0)	118 (35.9)	499 (27.0)	124 (40.7)
Diabetes, uncomplicated	809 (37.6)	127 (29.5)	305 (37.7)	199 (38.9)	33 (45.2)	145 (44.1)	658 (35.6)	151 (49.5)
Drug abuse	172 (8.0)	34 (7.9)	60 (7.4)	41 (8.0)	8 (11.0)	29 (8.8)	151 (8.2)	21 (6.9)
Fluid and electrolyte disorders	904 (42.0)	149 (34.7)	338 (41.8)	242 (47.4)	41 (56.2)	134 (40.7)	721 (39.0)	183 (60.0)
Hypertension, complicated	615 (28.6)	78 (18.1)	237 (29.3)	176 (34.4)	25 (34.2)	99 (30.1)	478 (25.9)	137 (44.9)
Hypertension, uncomplicated	1386 (64.4)	234 (54.4)	537 (66.4)	365 (71.4)	46 (63.0)	204 (62.0)	1133 (61.3)	253 (83.0)
Hypothyroidism	448 (20.8)	66 (15.3)	183 (22.6)	121 (23.7)	14 (19.2)	64 (19.5)	353 (19.1)	95 (31.1)
Liver disease	283 (13.2)	56 (13.0)	104 (12.9)	69 (13.5)	10 (13.7)	44 (13.4)	232 (12.6)	51 (16.7)
Lymphoma	29 (1.3)	6 (1.4)	10 (1.2)	10 (2.0)	73 (100.0)	3 (0.9)	23 (1.2)	6 (2.0)
Metastatic cancer	140 (6.5)	24 (5.6)	55 (6.8)	42 (8.2)	4 (5.5)	15 (4.6)	111 (6.0)	29 (9.5)
Obesity	682 (31.7)	112 (26.0)	264 (32.6)	161 (31.5)	29 (39.7)	116 (35.3)	582 (31.5)	100 (32.8)
Other neurological disorders	541 (25.1)	66 (15.3)	225 (27.8)	153 (29.9)	20 (27.4)	77 (23.4)	414 (22.4)	127 (41.6)
Paralysis	117 (5.4)	9 (2.1)	46 (5.7)	35 (6.8)	7 (9.6)	20 (6.1)	96 (5.2)	21 (6.9)
Peptic ulcer disease, excluding bleeding	113 (5.3)	22 (5.1)	46 (5.7)	32 (6.3)	2 (2.7)	11 (3.3)	90 (4.9)	23 (7.5)
Peripheral vascular disorders	521 (24.2)	69 (16.0)	200 (24.7)	155 (30.3)	25 (34.2)	72 (21.9)	400 (21.7)	121 (39.7)
Psychoses	206 (9.6)	32 (7.4)	80 (9.9)	67 (13.1)	7 (9.6)	20 (6.1)	166 (9.0)	40 (13.1)
Pulmonary circulation disorders	225 (10.5)	36 (8.4)	73 (9.0)	65 (12.7)	10 (13.7)	41 (12.5)	165 (8.9)	60 (19.7)
Renal failure	513 (23.8)	79 (18.4)	186 (23.0)	144 (28.2)	19 (26.0)	85 (25.8)	392 (21.2)	121 (39.7)
Rheumatoid arthritis/collagen vascular diseases	174 (8.1)	27 (6.3)	74 (9.1)	40 (7.8)	8 (11.0)	25 (7.6)	139 (7.5)	35 (11.5)
Solid tumor without metastasis	313 (14.5)	43 (10.0)	122 (15.1)	88 (17.2)	14 (19.2)	46 (14.0)	249 (13.5)	64 (21.0)
Valvular disease	527 (24.5)	74 (17.2)	229 (28.3)	141 (27.6)	17 (23.3)	66 (20.1)	420 (22.7)	107 (35.1)
Weight loss	356 (16.5)	59 (13.7)	140 (17.3)	107 (20.9)	8 (11.0)	42 (12.8)	277 (15.0)	79 (25.9)

Expired includes those that expired in the ED without known respiratory outcomes.

The majority of patients with respiratory outcomes did not require invasive ventilation (n = 1823, 84.7%). For these patients, male (49.8%) and female (50.2%) sex were similar in frequency with most frequently self-reported races of White (n = 908, 49.8%), Black (n = 444, 24.4%), and Other race (n = 398, 21.8%). The most prevalent comorbidities included uncomplicated hypertension (n = 1182, 64.8%), fluid & electrolyte disorders (n = 770, 42.2%), and cardiac arrhythmia (n = 694, 38.1%). Invasive ventilatory support was required for 15.3% (n = 329) of patients with respiratory outcomes. The majority of those who required invasive ventilatory support were male (n = 215, 65.3%) with self-reported race of White (n = 113, 34.3%), Black (n = 100, 30.4%), and Other race (n = 99, 30.1%). The most prevalent comorbidities included uncomplicated hypertension (n = 204, 62.0%), diabetes without complication (n = 145, 44.1%), and fluid & electrolyte disorders (n = 134, 40.7%).

**Fig 2:**
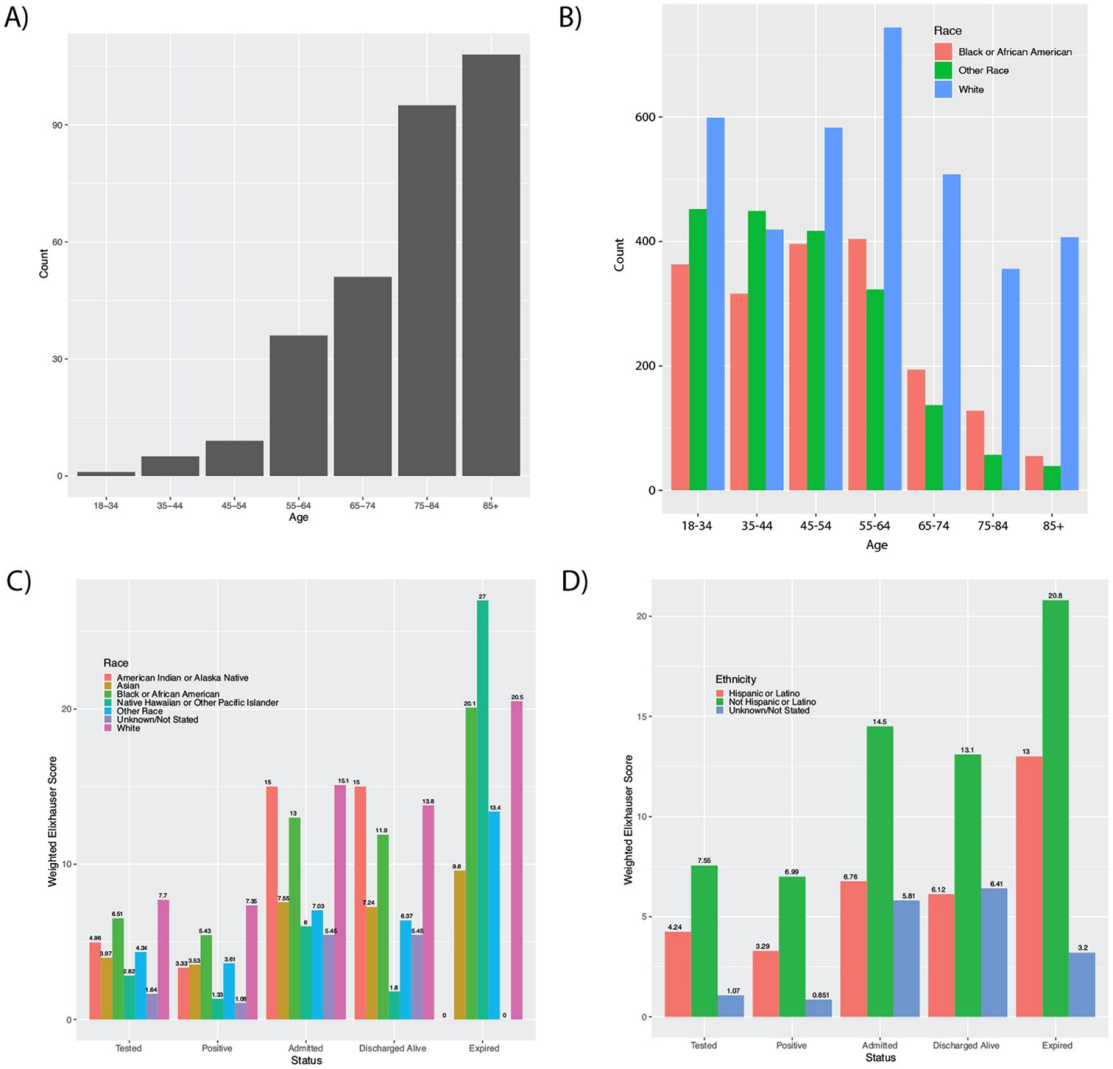
A) Frequency of in-hospital mortality by age, B) distribution of age by self-reported race in patients positive for SARS-CoV-2, and weighted Elixhauser comorbidity scores by patient status grouped by C) recorded race and D) recorded ethnicity.

In-hospital mortality was 14.2% (n = 305) of patients with a discharge disposition and these patients had a median length of stay of 7.9 days (IQR 3.5–15.1). The majority of patients who experienced in-hospital mortality were male (n = 175, 57.4%) and mortality increased with age (Fig 2A); the median age of those who experienced in-hospital mortality was 80.7 (IQR 70.5–88.6) years. Those with older age, particularly those ≥85 years old, predominantly self-reported a race of White (Fig 2B). The comorbidities most common among those who expired were uncomplicated hypertension (n = 253, 83.0%), fluid & electrolyte disorders (n = 183, 60.0%), and cardiac arrhythmia (n = 162, 53.1%). Those who were admitted and/or experienced in-hospital mortality compared to those who tested positive for SARS-CoV-2 in all racial and ethnic groups had an increased comorbidity burden as determined by weighted Elixhauser comorbidity scores (Fig 2C and 2D), with the exception of those with Unknown ethnicity which represented a small number of patients. For those who expired, the most common recorded races were White (n = 185, 60.7%), Black (n = 69, 22.6%), and Other race (n = 43, 14.1%). Those who reported Hispanic ethnicity accounted for 16.1% (n = 49) of in-hospital mortality. In-hospital, age-adjusted mortality rates were 4.1%, 3.8%, 5.3%, 4.0%, and 4.3% for those who reported a race of White, Black, Asian, Hawaiian or Pacific Islander, and Other race, respectively (S3 Fig). Those who reported Hispanic ethnicity had an age-adjusted in-hospital mortality rate of 4.4%.

**Fig 3:**
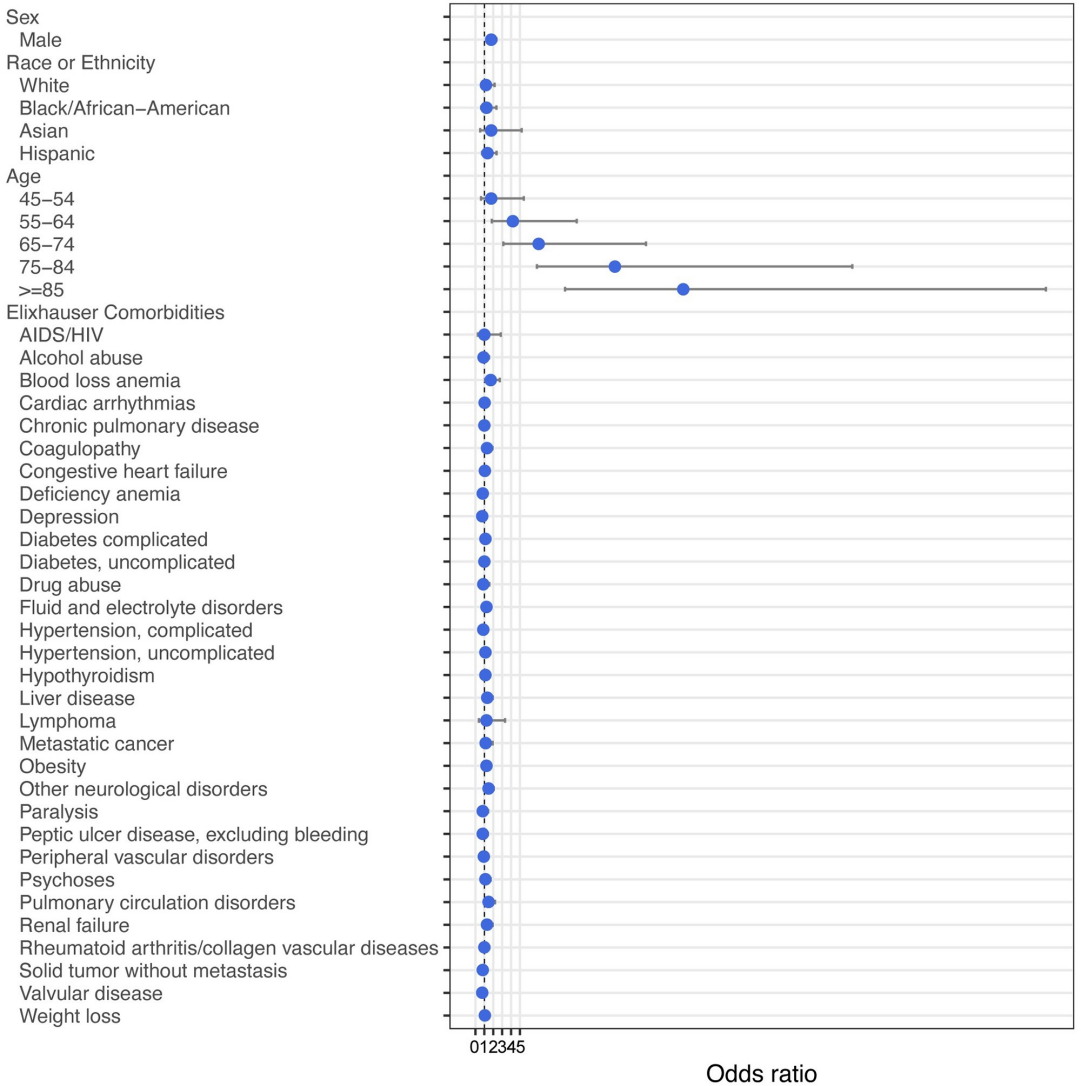
Multivariable analysis with odds ratios for mortality in discharged patients. Bars indicate 95% confidence intervals.

As seen with admission, regression analysis demonstrated that increased age had the highest risk for in-hospital mortality (Fig 3, S3 Table), with the largest risk seen for those ≥85 years old (OR 23.3, 95%CI = 10.1–64.1). Male sex was also associated with increased odds of in-hospital mortality (OR 1.76, 95%CI = 1.33–2.35). Of the comorbidities present within the medical history and problem list of the EHR, only a history of blood loss anemia (OR 1.72, 95%CI = 1.07–2.74) and other neurological disorders (OR 1.47, 95%CI = 1.06–2.05) were significant. Race was not statistically associated with a risk of in-hospital mortality in this cohort.

### Treatment pathways for admitted patients with Covid-19

**Fig 4:**
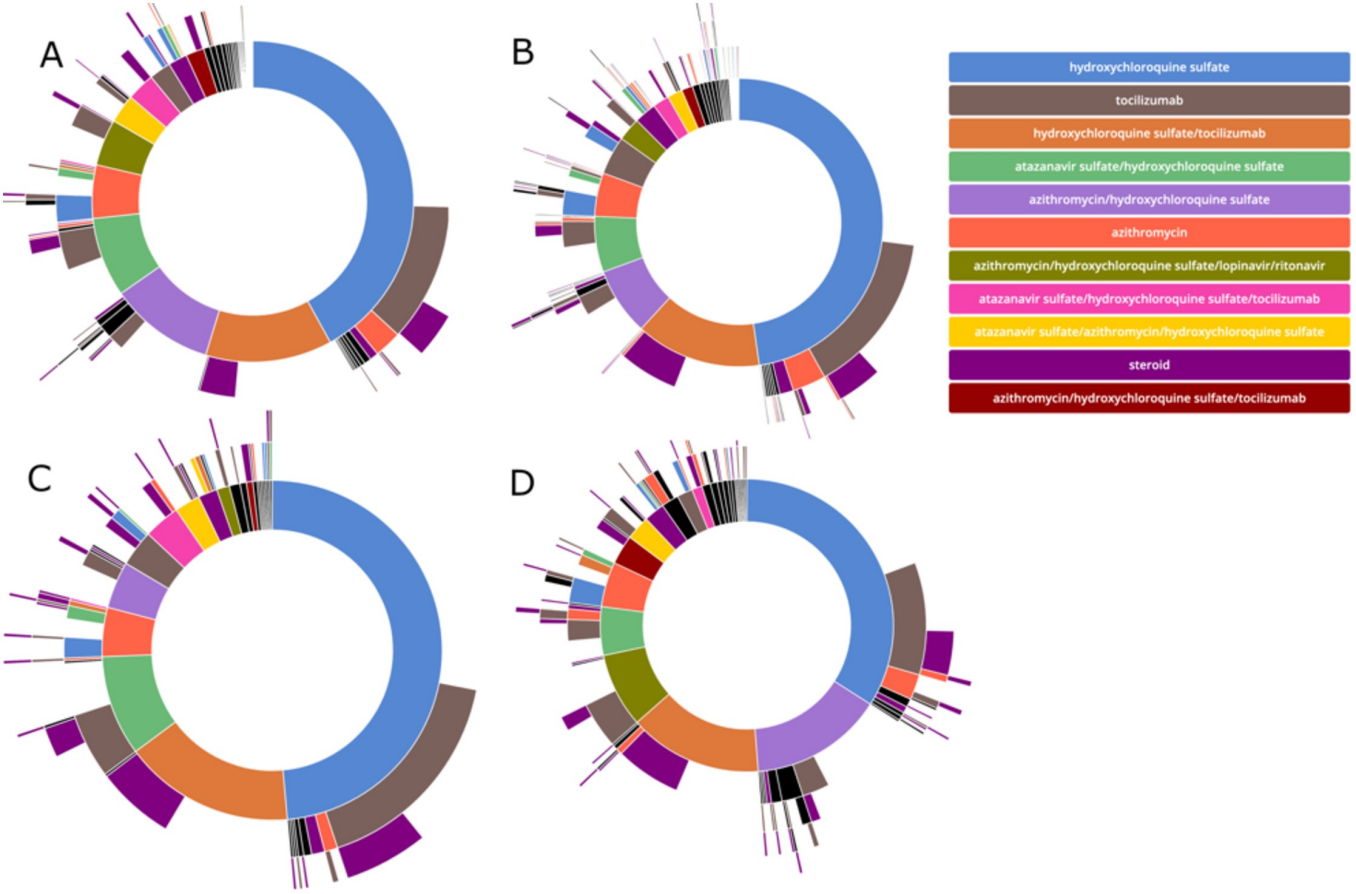
Sunburst diagram of medication pathways with individual regimens grouped by order of initiation. Individual plots show the treatment pathways for A) all patients and those with a recorded race of B) White, C) Black, and D) Other.

Of patients with known outcomes, 1895 (88.1%) received medications for Covid-19 treatment while admitted. We assessed treatment pathways for 13 Covid-19 related medications. Patients were treated with 188 different possible medication regimen permutations with 50 unique combinations (Fig 4, S4 Table). The most common first line regimens included hydroxychloroquine (88.3% of patients), tocilizumab (23.8%) and azithromycin (22.7%). The most frequent second-line regimens, aside from the most frequent first line agents, included the addition of steroids (21.3%), atazanavir (6.5%), and lopinavir/ritonavir (3.4%). The most common treatment permutations were hydroxychloroquine alone (25.2%), hydroxychloroquine in combination with tocilizumab (18.8%), or hydroxychloroquine in combination with azithromycin (8.1%). A total of just six Covid-19 related medications were given to more than 1% of admitted patients in our cohort: hydroxychloroquine sulfate (94.7%), tocilizumab (51.0%), azithromycin (28.8%), steroids (24.3%), atazanavir (15.5%), and lopinavir/ritonavir (7.8%). All race and ethnicity groups were prescribed hydroxychloroquine most frequently, with patients who self-reported as Asian having the lowest rate (92.7%). Tocilizumab was the second most frequently prescribed medication in all groups. The use of azithromycin had the most notable variation among groups: it was the second most common medication in those identifying as Other race, with a frequency of 46.7% of patients, but was fifth most common among those who identified as Black, with only 16.7% of patients receiving azithromycin.

## Discussion

In one of the largest real-world analyses of risk factors associated with Covid-19 infection and disease severity, we identified age as the primary risk factor associated with both admission and in-hospital mortality in those infected with SARS-CoV-2. Black race and Hispanic ethnicity were associated with increased risk of admission in our cohort and had increased disease and mortality burden, but age-adjusted in-hospital mortality was similar among all reported races and ethnicities. Comorbidities had much less impact on risk for either admission or in-hospital mortality in our study.

Our work extends the literature in several important ways. Firstly, we followed a single large cohort to identify risks associated with infection and severe disease from the time of testing through discharge. Secondly, we provide further evidence that age and male sex are significantly associated risk factors for both admission and in-hospital mortality. Thirdly, we found that comorbidities, while common in those with SARS-CoV-2, were not strongly associated with either admission or in-hospital mortality based on multivariable analysis. Fourthly, we found race and ethnicity to be associated with infection and admission in this cohort, but with in-hospital mortality that was similar among these groups in our discharged population. Finally, we identified consistent use of medications within our admitted population, but with many possible treatment pathways for any individual patient and with frequent use of investigational therapies for Covid-19. Further investigation is needed to characterize potential benefits or risks associated with the various treatment pathways that have been available over the course of the pandemic.

Our data confirm findings in other studies that show age as a primary risk factor for admission and in-hospital mortality in adult patients and that male sex is also highly associated with these outcomes [5, 7, 23–26]. While the mechanisms that may lead to more severe disease in men have not been definitively elucidated, several potential mechanisms have been proposed to explain the demonstrated differences, including increased comorbidities and changes in the immune response in those who are male and older, along with possible genetic/biologic differences that may increase disease severity in men [27]. Similarly, immune senescence, with dysregulated inflammation and decreased adaptive immune response, has been hypothesized as a possible reason for worse disease in older populations [28].

Many studies have shown that Covid-19 has disproportionately affected minority populations across the US [29–32]. Within our cohort, Black and Hispanic populations were overrepresented in those who were tested, positive, and admitted for SARS-CoV-2 compared to census data for Connecticut. Studies based on regional mortality data, which have included out-of-hospital mortality, have shown that severe disease may also be more prevalent in minority populations [10, 30]. In our study, Black race was overrepresented in those with more severe outcomes compared to state census numbers. However, in the discharged population, we found that age-adjusted, in-hospital mortality was similar among all racial and ethnic groups, with rates ranging from 3.8% to 5.3%. This finding is consistent with other studies of in-hospital mortality related to Covid-19 [3, 23], but also demonstrates that minority populations experience a higher overall burden of disease. While a small percentage of this cohort did not have race or ethnicity data provided, it remains limited by the potential for errors during patient registration and the possibility of provider-reported responses.

Our data reflect the prominence of comorbidities in those with SARS-CoV-2 infection. While comorbidities were common, some of the most commonly reported risks for severe disease [2, 33, 34] were not identified as risks in this study and multivariable analysis did not find a history of hypertension or diabetes to be significantly associated with admission. An increased comorbidity burden was noted in those with in-hospital mortality compared to those who were discharged alive. However, multivariable analysis only identified a history of blood loss anemia to be significantly associated with in-hospital mortality. Other comorbidities, such as obesity, were associated with admission but not in-hospital mortality. It is unclear if these patients required admission due to more severe disease or were admitted due to perceived risk based on early reports of Covid-19 risk factors. Similarly, a history of drug abuse and psychoses were associated with admission, but likely represented more frequent testing in these populations with limited ability to discharge patients to shared facilities following a positive SARS-CoV-2 test. These findings highlight the fact that age and sex appear to be the predominant drivers of severe disease. Additional studies will be needed to further characterize the risk of underlying disease on the severity of Covid-19.

The risks and outcomes reported here should be assessed in the context of the treatment protocols used during this period of the epidemic. Treatment standards based on early recommendations led to a majority of patients receiving disease related therapy, often with investigational treatments. Of patients who received a Covid-19 targeted therapy, 94.7% received hydroxychloroquine, 51.0% received tocilizumab, and 28.8% received azithromycin. The use of Covid-19 directed treatments was consistent among races and ethnicities in our cohort. But despite an early push to use promising medications from in vitro studies, such as hydroxychloroquine and azithromycin, evidence now demonstrates that neither is likely beneficial for admitted patients. As such, the treatment context of future studies should be similarly assessed to determine whether changes in treatment pathways impact the reported risks and outcomes in those with Covid-19.

Our analysis leveraged real-world data derived from the EHR to assess all patients tested for SARS-CoV-2 within our health system. We implemented computed phenotypes to identify cases and clinically relevant outcomes, with a subset manually reviewed for accuracy. Our findings add to a growing base of evidence related to Covid-19 risk factors and outcomes. However, as an observational study based on real-world data, this study also has several limitations. First, while standardized testing protocols were in place, testing was often limited to symptomatic individuals or those with known exposure risks, thus potentially biasing our cohort to those who were symptomatic and sought care. The study was also limited to a single health system, but one that consists of a mixture of academic, urban, and suburban care facilities with a diverse patient population. In addition, while our health system implemented standardized treatment protocols, patients received therapies that were investigational for Covid-19 at the time of the study and use of these medications may not be similar at all institutions, especially as Covid-19 treatment protocols rapidly evolve as new evidence is obtained. Another limitation is that features associated with risk of admission may not correlate to risk of disease severity, as the decision to admit can be impacted based on discharge options or perceived clinical risks by healthcare providers. Finally, due to the timeline of the current outbreak, this study was limited to the initial admission and only assessed in-hospital mortality. Therefore, additional studies are needed to assess the impact of disease on patients not admitted to the hospital and the long-term effects of SARS-CoV-2 infection.

There is an ongoing need to rapidly generate and communicate evidence, while also being cautious that only high-quality data are used to inform policy and develop clinical recommendations. While waiting for larger, more comprehensive case control and population-scale studies to define COVID-19 specific risks, prevalence, treatment, and outcomes, providers and public health officials need the best available evidence for clinical use. The data presented here provide findings from a large cohort that was followed from testing through discharge, identified increased age and male sex as the strongest risk factors for admission and in-hospital mortality, and found that in-hospital mortality was similar in racial and ethnic groups within our health system. Ongoing studies that further elucidate the risk of comorbidities, particularly given rapidly evolving treatment guidelines, remain needed as the Covid-19 pandemic continues to grow.

## Conclusion

The early COVID-19 experience at YNHH demonstrated that increasing age and male sex are the risks most strongly associated with admission and in-hospital mortality in those with SARS-CoV-2 infection. Minority racial and ethnic groups had increased risk of admission and higher disease burden, including mortality. But, for discharged patients, in-hospital mortality rates were similar in all racial and ethnic groups. While comorbidities were frequently observed in patients with SARS-CoV-2, few were associated with admission or in-hospital mortality in our cohort. Despite the limitations, this dataset from a multi-hospital health system with a diverse patient population presents valuable information related to risk factors for SARS-CoV-2 infection and short-term outcomes.

## Supporting information

S1 Fig Patient counts and exclusions based on computed phenotyping criteria. (DOCX)

S2 Fig Cumulative patients tested (blue) and positive (red) for SARS-CoV-2. (DOCX)

S3 Fig In-hospital, age-adjusted mortality in discharged patients with SARS-CoV-2. (DOCX)

S1 Table Race and ethnicity as noted in the EHR and mapped to the OMOP CDM. (DOCX)

S2 Table Multivariable analysis with odds ratios for admission in patients with a positive SARS-CoV-2 test compared to patients who were not admitted. (DOCX)

S3 Table Multivariable analysis with odds ratios for mortality in discharged patients. (DOCX)

S4 Table Frequency of each medication pathway used in patients who received at least 1 Covid-19 directed therapy. Those categories with less than 4 patients were reported as ≤3.
(DOCX)

## References

[1] Dong E, Du H, Gardner L. An interactive web-based dashboard to track COVID-19 in real time. Lancet Infect Dis. 2020;20: 533–534. 10.1016/S1473-3099(20)30120-1 32087114 PMC7159018

[2] Richardson S, Hirsch JS, Narasimhan M, Crawford JM, McGinn T, Davidson KW, et al. Presenting Characteristics, Comorbidities, and Outcomes Among 5700 Patients Hospitalized With COVID-19 in the New York City Area. JAMA. 2020; 10.1001/jama.2020.6775 32320003 PMC7177629

[3] Suleyman G, Fadel RA, Malette KM, Hammond C, Abdulla H, Entz A, et al. Clinical characteristics and morbidity associated with coronavirus disease 2019 in a series of patients in metropolitan detroit. JAMA Netw Open. 2020;3: e2012270. 10.1001/jamanetworkopen.2020.12270 32543702 PMC7298606

[4] Anesi GL, Halpern SD, Delgado MK. Covid-19 related hospital admissions in the United States: needs and outcomes. BMJ. 2020;369: m2082. 10.1136/bmj.m2082 32461214

[5] Williamson EJ, Walker AJ, Bhaskaran K, Bacon S, Bates C, Morton CE, et al. OpenSAFELY: factors associated with COVID-19 death in 17 million patients. Nature. 2020; 10.1038/s41586-020-2521-4 32640463 PMC7611074

[6] Verity R, Okell LC, Dorigatti I, Winskill P, Whittaker C, Imai N, et al. Estimates of the severity of coronavirus disease 2019: a model-based analysis. Lancet Infect Dis. 2020;20: 669–677. 10.1016/S1473-3099(20)30243-7 32240634 PMC7158570

[7] Onder G, Rezza G, Brusaferro S. Case-Fatality Rate and Characteristics of Patients Dying in Relation to COVID-19 in Italy. JAMA. 2020 3 23; 10.1001/jama.2020.4683 32203977

[8] Chen F, Sun W, Sun S, Li Z, Wang Z, Yu L. Clinical characteristics and risk factors for mortality among inpatients with COVID-19 in Wuhan, China. Clin Transl Med. 2020; 10.1002/ctm2.40 32508024 PMC7300688

[9] Zhou F, Yu T, Du R, Fan G, Liu Y, Liu Z, et al. Clinical course and risk factors for mortality of adult inpatients with COVID-19 in Wuhan, China: a retrospective cohort study. The Lancet. 2020 3;395(10229):1054–62. 10.1016/S0140-6736(20)30566-3 32171076 PMC7270627

[10] Wadhera RK, Wadhera P, Gaba P, Figueroa JF, Joynt Maddox KE, Yeh RW, et al. Variation in COVID-19 Hospitalizations and Deaths Across New York City Boroughs. JAMA. 2020; 10.1001/jama.2020.7197 32347898 PMC7191469

[11] Bialek S, Bowen V, Chow N, Curns A, Gierke R, et al. Geographic Differences in COVID-19 Cases, Deaths, and Incidence—United States, February 12–April 7, 2020. MMWR Morbidity and Mortality Weekly Report. 2020 4 17;69(15):465–71. 10.15585/mmwr.mm6915e4 32298250 PMC7755058

[12] Yale New Haven Health | facts and figures [Internet]. [cited 16 Jul 2020]. https://www.ynhh.org/ynhhs/about/corporate-overview/system-statistics

[13] OMOP Common Data Model–OHDSI [Internet]. [cited 2 Mar 2019]. https://www.ohdsi.org/data-standardization/the-common-data-model/

[14] McPadden J, Durant TJ, Bunch DR, Coppi A, Price N, Rodgerson K, et al. Health care and precision medicine research: analysis of a scalable data science platform. J Med Internet Res. 2019;21: e13043. 10.2196/13043 30964441 PMC6477571

[15] Schulz WL, Durant TJS, Torre CJ, Hsiao AL, Krumholz HM. Agile Health Care Analytics: Enabling Real-Time Disease Surveillance With a Computational Health Platform. J Med Internet Res. 2020;22: e18707. 10.2196/18707 32442130 PMC7257473

[16] Bostock M, Ogievetsky V, Heer J. D3: Data-Driven Documents. IEEE Trans Vis Comput Graph. 2011;17: 2301–2309. 10.1109/TVCG.2011.185 22034350

[17] Elixhauser A, Steiner C, Harris DR, Coffey RM. Comorbidity measures for use with administrative data. Med Care. 1998;36: 8–27. 10.1097/00005650-199801000-00004 9431328

[18] Gasparini A. comorbidity: An R package for computing comorbidity scores. JOSS. 2018;3: 648. 10.21105/joss.00648

[19] Moore BJ, White S, Washington R, Coenen N, Elixhauser A. Identifying Increased Risk of Readmission and In-hospital Mortality Using Hospital Administrative Data: The AHRQ Elixhauser Comorbidity Index. Med Care. 2017;55: 698–705. 10.1097/MLR.0000000000000735 28498196

[20] van Walraven C, Austin PC, Jennings A, Quan H, Forster AJ. A modification of the Elixhauser comorbidity measures into a point system for hospital death using administrative data. Med Care. 2009;47: 626–633. 10.1097/MLR.0b013e31819432e5 19433995

[21] Klein RJ, Schoenborn CA. Age adjustment using the 2000 projected U.S. population. Healthy People 2010 Stat Notes. 2001; 1–10. 11676466

[22] U.S. Census Bureau QuickFacts: Connecticut [Internet]. [cited 16 Jul 2020]. https://www.census.gov/quickfacts/CT

[23] Petrilli CM, Jones SA, Yang J, Rajagopalan H, O’Donnell L, Chernyak Y, et al. Factors associated with hospital admission and critical illness among 5279 people with coronavirus disease 2019 in New York City: prospective cohort study. BMJ. 2020 5 22;m1966. 10.1136/bmj.m1966 32444366 PMC7243801

[24] Zhou F, Yu T, Du R, Fan G, Liu Y, Liu Z, et al. Clinical course and risk factors for mortality of adult inpatients with COVID-19 in Wuhan, China: a retrospective cohort study. The Lancet. 2020 3;395(10229):1054–62. 10.1016/S0140-6736(20)30566-3 32171076 PMC7270627

[25] Jin J-M, Bai P, He W, Wu F, Liu X-F, Han D-M, et al. Gender Differences in Patients With COVID-19: Focus on Severity and Mortality. Frontiers in Public Health. 2020 4 29;8. 10.3389/fpubh.2020.00152 32411652 PMC7201103

[26] Castelnuovo A, Bonaccio M, Costanzo S, Gialluisi A, Antinori A, Berselli N, et al. Common cardiovascular risk factors and in-hospital mortality in 3,894 patients with COVID-19: survival analysis and machine learning-based findings from the multicentre Italian CORIST Study. Nutr Metab Cardiovasc Dis. 2020 10 30;30(11):1899–1913. 10.1016/j.numecd.2020.07.031 32912793 PMC7833278

[27] Takahashi T, Wong P, Ellingson M, Lucas C, Klein J, Israelow B, et al. Sex differences in immune responses to SARS-CoV-2 that underlie disease outcomes. medRxiv; 2020. 10.1101/2020.06.06.20123414 32577695 PMC7302304

[28] Chen J, Kelley WJ, Goldstein DR. Role of Aging and the Immune Response to Respiratory Viral Infections: Potential Implications for COVID-19. J Immunol. 2020;205: 313–320. 10.4049/jimmunol.2000380 32493812 PMC7343582

[29] Abedi V, Olulana O, Avula V, Chaudhary D, Khan A, Shahjouei S, et al. Racial, Economic and Health Inequality and COVID-19 Infection in the United States. medRxiv; 2020. 10.1101/2020.04.26.20079756 32875535 PMC7462354

[30] Gross CP, Essien UR, Pasha S, Gross JR, Wang S, Nunez-Smith M. Racial and Ethnic Disparities in Population Level Covid-19 Mortality. medRxiv. 2020; 10.1007/s11606-020-06081-w 32754782 PMC7402388

[31] Price-Haywood EG, Burton J, Fort D, Seoane L. Hospitalization and Mortality among Black Patients and White Patients with COVID-19. N Engl J Med. 2020;382: 2534–2543. 10.1056/NEJMsa2011686 32459916 PMC7269015

[32] Azar KMJ, Shen Z, Romanelli RJ, Lockhart SH, Smits K, Robinson S, et al. Disparities In Outcomes Among COVID-19 Patients In A Large Health Care System In California. Health Aff (Millwood). 2020;39: 1253–1262. 10.1377/hlthaff.2020.00598 32437224

[33] Kass DA, Duggal P, Cingolani O. Obesity could shift severe COVID-19 disease to younger ages. Lancet. 2020;395: 1544–1545. 10.1016/S0140-6736(20)31024-2 32380044 PMC7196905

[34] Gao C, Cai Y, Zhang K, Zhou L, Zhang Y, Zhang X, et al. Association of hypertension and antihypertensive treatment with COVID-19 mortality: a retrospective observational study. Eur Heart J. 2020;41: 2058–2066. 10.1093/eurheartj/ehaa433 32498076 PMC7314067

