# Supplementary figures and images for: Clinical characteristics and outcomes for 7,995 patients with SARS-CoV-2 infection
Source: PLoS One. 2021 Mar 31;16(3):e0243291. doi: 10.1371/journal.pone.0243291 (PMC8011821; doi:10.1371/journal.pone.0243291)

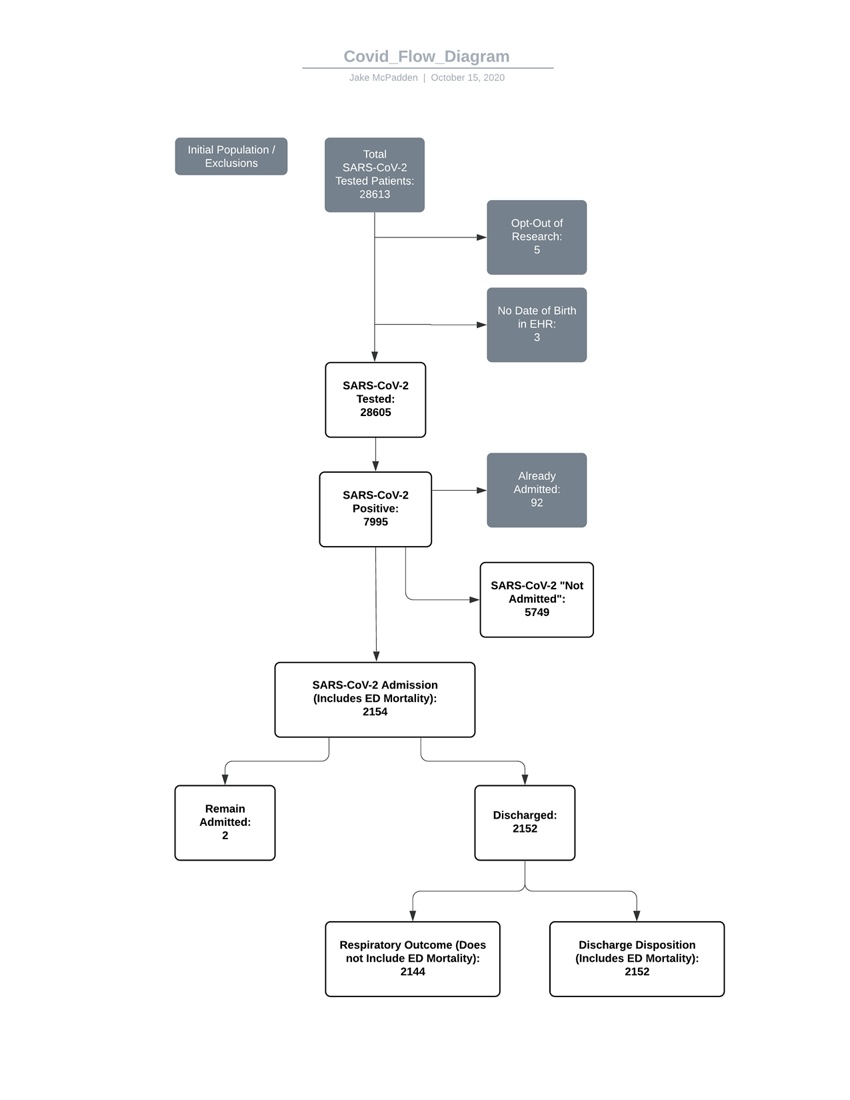


S1 Fig. Patient counts and exclusions based on computed phenotyping criteria.

Supplement: S1 Fig — (DOCX) [file pone.0243291.s001.docx]

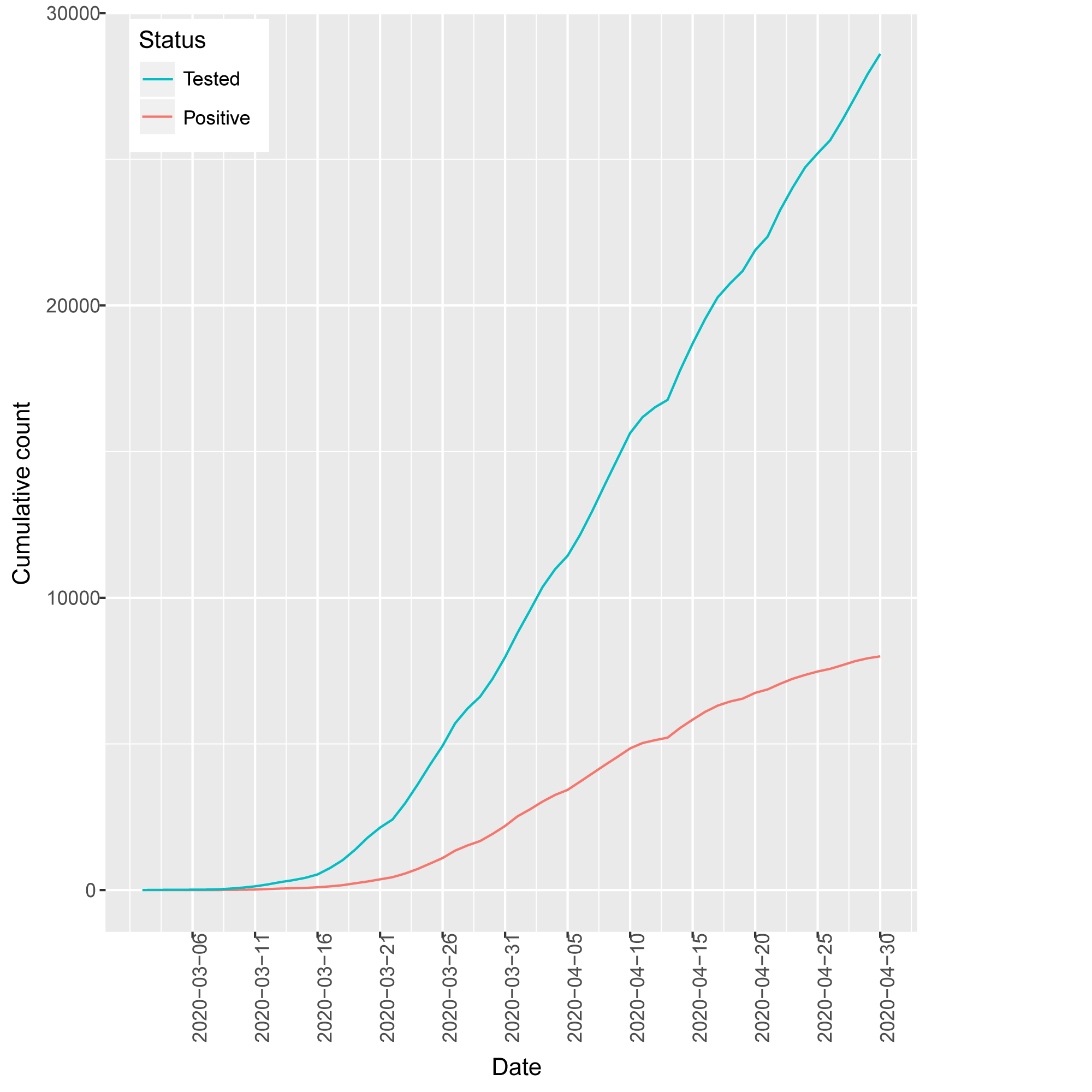


S2 Fig. Cumulative patients tested (blue) and positive (red) for SARS-CoV-2.

Supplement: S2 Fig — (DOCX) [file pone.0243291.s002.docx]

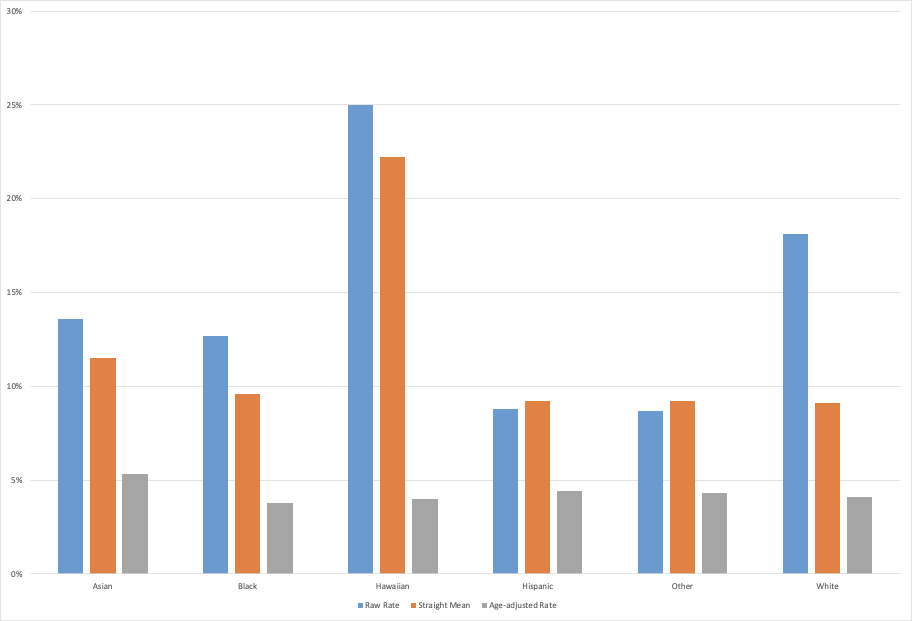


S3 Fig. In-hospital, age-adjusted mortality in discharged patients with SARS-CoV-2.

Supplement: S3 Fig — (DOCX) [file pone.0243291.s003.docx]
