# Supplementary material for: Clinical characteristics and outcomes for 7,995 patients with SARS-CoV-2 infection
Source: PLoS One. 2021 Mar 31;16(3):e0243291. doi: 10.1371/journal.pone.0243291 (PMC8011821; doi:10.1371/journal.pone.0243291)
Supplement: S1 Table — (DOCX) [file pone.0243291.s004.docx]

S1 Table. Race and ethnicity as noted in the EHR and mapped to the OMOP CDM.

| **EHR-Recorded Race or Ethnicity** | **OMOP Mapping** | **Abbreviation** |
| --- | --- | --- |
| American Indian or Alaska Native | American Indian or Alaska Native | American Indian or Alaska Native |
| Asian | Asian | Asian |
| Black or African American | Black or African American | Black |
| Native Hawaiian | Native Hawaiian or Other Pacific Islander | Native Hawaiian or Other Pacific Islander |
| Other Pacific Islander | Native Hawaiian or Other Pacific Islander | Native Hawaiian or Other Pacific Islander |
| Other/Not Listed | Other | Other |
| Patient Refused | Unknown/Not Stated | Unknown/Not Stated |
| Unknown | Unknown/Not Stated | Unknown/Not Stated |
| White or Caucasian | White | White |
| Hispanic or Latino | Hispanic or Latino | Hispanic |
| Not Hispanic or Latino | Not Hispanic or Latino | Not Hispanic |
