# Supplementary material for: Clinical characteristics and outcomes for 7,995 patients with SARS-CoV-2 infection
Source: PLoS One. 2021 Mar 31;16(3):e0243291. doi: 10.1371/journal.pone.0243291 (PMC8011821; doi:10.1371/journal.pone.0243291)
Supplement: S2 Table — (DOCX) [file pone.0243291.s005.docx]

S2 Table. Multivariable analysis with odds ratios for admission in patients with a positive SARS-CoV-2 test compared to patients who were not admitted.

|  | Odds Ratio | CI 2.50% | CI 97.50% | p |
| --- | --- | --- | --- | --- |
| (Intercept) | 0.052 | 0.040 | 0.067 | <0.01 |
| Sex |  |  |  |  |
| Male | 1.68 | 1.48 | 1.90 | <0.01 |
| Race or Ethnicity |  |  |  |  |
| American Indian | 0.44 | 0.02 | 2.68 | 0.47 |
| Asian | 1.58 | 1.02 | 2.41 | 0.04 |
| Black | 1.43 | 1.14 | 1.78 | <0.01 |
| Hawaiian/Pacific Islander | 1.45 | 0.53 | 3.52 | 0.44 |
| Hispanic | 1.81 | 1.50 | 2.18 | <0.01 |
| White | 0.85 | 0.70 | 1.03 | 0.09 |
| Age |  |  |  |  |
| 35-44 | 1.43 | 1.13 | 1.81 | <0.01 |
| 45-54 | 1.76 | 1.41 | 2.21 | <0.01 |
| 55-64 | 3.24 | 2.60 | 4.04 | <0.01 |
| 65-74 | 6.95 | 5.45 | 8.91 | <0.01 |
| 75-84 | 15.91 | 11.92 | 21.33 | <0.01 |
| >85 | 22.03 | 16.10 | 30.30 | <0.01 |
| Elixhauser Comorbidities |  |  |  |  |
| AIDS/HIV | 1.26 | 0.71 | 2.20 | 0.43 |
| Alcohol abuse | 1.24 | 0.95 | 1.62 | 0.12 |
| Blood loss anemia | 0.98 | 0.70 | 1.35 | 0.88 |
| Cardiac arrhythmias | 1.13 | 0.96 | 1.33 | 0.13 |
| Chronic pulmonary disease | 0.94 | 0.81 | 1.09 | 0.43 |
| Coagulopathy | 1.11 | 0.87 | 1.43 | 0.39 |
| Congestive heart failure | 1.06 | 0.83 | 1.36 | 0.63 |
| Deficiency anemia | 0.82 | 0.67 | 1.00 | 0.05 |
| Depression | 0.85 | 0.72 | 1.01 | 0.06 |
| Diabetes, complicated | 1.18 | 0.94 | 1.47 | 0.15 |
| Diabetes, uncomplicated | 1.16 | 0.95 | 1.40 | 0.15 |
| Drug abuse | 1.46 | 1.11 | 1.92 | 0.01 |
| Fluid and electrolyte disorders | 1.99 | 1.67 | 2.37 | <0.01 |
| Hypertension, complicated | 1.14 | 0.88 | 1.48 | 0.31 |
| Hypertension, uncomplicated | 0.97 | 0.83 | 1.13 | 0.68 |
| Hypothyroidism | 0.89 | 0.75 | 1.06 | 0.21 |
| Liver disease | 1.01 | 0.83 | 1.23 | 0.91 |
| Lymphoma | 1.07 | 0.58 | 1.92 | 0.83 |
| Metastatic cancer | 1.55 | 1.11 | 2.15 | 0.01 |
| Obesity | 1.18 | 1.02 | 1.37 | 0.02 |
| Other neurological disorders | 1.31 | 1.07 | 1.61 | 0.01 |
| Paralysis | 1.00 | 0.70 | 1.44 | 1.00 |
| Peptic ulcer disease, excluding bleeding | 1.47 | 1.04 | 2.07 | 0.03 |
| Peripheral vascular disorders | 0.84 | 0.68 | 1.04 | 0.12 |
| Psychoses | 1.98 | 1.47 | 2.69 | <0.01 |
| Pulmonary circulation disorders | 1.53 | 1.14 | 2.06 | 0.01 |
| Renal failure | 1.38 | 1.08 | 1.75 | 0.01 |
| Rheumatoid arthritis/collagen vascular diseases | 0.89 | 0.69 | 1.15 | 0.38 |
| Solid tumor without metastasis | 0.87 | 0.69 | 1.09 | 0.22 |
| Valvular disease | 1.01 | 0.83 | 1.23 | 0.93 |
| Weight loss | 0.98 | 0.78 | 1.23 | 0.88 |
