# Supplementary material for: Clinical characteristics and outcomes for 7,995 patients with SARS-CoV-2 infection
Source: PLoS One. 2021 Mar 31;16(3):e0243291. doi: 10.1371/journal.pone.0243291 (PMC8011821; doi:10.1371/journal.pone.0243291)
Supplement: S3 Table — (DOCX) [file pone.0243291.s006.docx]

S3 Table. Multivariable analysis with odds ratios for mortality in discharged patients.

|  | Odds Ratio | CI 2.50% | CI 97.50% | p |
| --- | --- | --- | --- | --- |
| (Intercept) | 0.008 | 0.003 | 0.021 | <0.001 |
| Sex |  |  |  |  |
| Male | 1.76 | 1.33 | 2.35 | <0.01 |
| Race or Ethnicity |  |  |  |  |
| Asian | 1.76 | 0.54 | 5.18 | 0.32 |
| Black/African-American | 1.22 | 0.64 | 2.36 | 0.55 |
| Hispanic | 1.34 | 0.75 | 2.37 | 0.32 |
| White | 1.18 | 0.65 | 2.14 | 0.59 |
| Age |  |  |  |  |
| 45-54 | 1.78 | 0.63 | 5.42 | 0.28 |
| 55-64 | 4.20 | 1.83 | 11.38 | <0.01 |
| 65-74 | 7.10 | 3.13 | 19.16 | <0.01 |
| 75-84 | 15.66 | 6.91 | 42.34 | <0.01 |
| >85 | 23.34 | 10.06 | 64.09 | <0.01 |
| Elixhauser Comorbidities |  |  |  |  |
| AIDS/HIV | 1.00 | 0.27 | 2.84 | 0.99 |
| Alcohol abuse | 0.91 | 0.52 | 1.53 | 0.73 |
| Blood loss anemia | 1.72 | 1.07 | 2.74 | 0.02 |
| Cardiac arrhythmias | 1.03 | 0.74 | 1.43 | 0.85 |
| Chronic pulmonary disease | 1.01 | 0.74 | 1.39 | 0.93 |
| Coagulopathy | 1.31 | 0.89 | 1.92 | 0.16 |
| Congestive heart failure | 1.06 | 0.71 | 1.56 | 0.79 |
| Deficiency anemia | 0.83 | 0.58 | 1.20 | 0.33 |
| Depression | 0.78 | 0.56 | 1.08 | 0.14 |
| Diabetes, complicated | 1.10 | 0.72 | 1.68 | 0.66 |
| Diabetes, uncomplicated | 0.99 | 0.66 | 1.47 | 0.96 |
| Drug abuse | 0.89 | 0.49 | 1.55 | 0.68 |
| Fluid and electrolyte disorders | 1.22 | 0.86 | 1.72 | 0.27 |
| Hypertension, complicated | 0.88 | 0.56 | 1.36 | 0.56 |
| Hypertension, uncomplicated | 1.10 | 0.73 | 1.67 | 0.65 |
| Hypothyroidism | 1.12 | 0.81 | 1.54 | 0.51 |
| Liver disease | 1.32 | 0.88 | 1.94 | 0.17 |
| Lymphoma | 1.21 | 0.39 | 3.32 | 0.72 |
| Metastatic cancer | 1.15 | 0.67 | 1.93 | 0.61 |
| Obesity | 1.22 | 0.89 | 1.69 | 0.22 |
| Other neurological disorders | 1.47 | 1.06 | 2.05 | 0.02 |
| Paralysis | 0.83 | 0.47 | 1.42 | 0.51 |
| Peptic ulcer disease, excluding bleeding | 0.82 | 0.46 | 1.40 | 0.47 |
| Peripheral vascular disorders | 0.94 | 0.67 | 1.33 | 0.74 |
| Psychoses | 1.11 | 0.71 | 1.70 | 0.65 |
| Pulmonary circulation disorders | 1.45 | 0.96 | 2.19 | 0.07 |
| Renal failure | 1.31 | 0.89 | 1.93 | 0.16 |
| Rheumatoid arthritis/collagen vascular diseases | 1.01 | 0.64 | 1.55 | 0.96 |
| Solid tumor without metastasis | 0.84 | 0.57 | 1.21 | 0.35 |
| Valvular disease | 0.75 | 0.53 | 1.06 | 0.11 |
| Weight loss | 1.04 | 0.73 | 1.48 | 0.83 |
