# Supplementary material for: Clinical characteristics and outcomes for 7,995 patients with SARS-CoV-2 infection
Source: PLoS One. 2021 Mar 31;16(3):e0243291. doi: 10.1371/journal.pone.0243291 (PMC8011821; doi:10.1371/journal.pone.0243291)
Supplement: S4 Table — Those categories with less than 4 patients were reported as ≤3. (DOCX) [file pone.0243291.s007.docx]

S4 Table. Frequency of each medication pathway used in patients who received at least 1 Covid-19 directed therapy. Those categories with less than 4 patients were reported as ≤3.

| Medications Grouped by Date of Treatment Initiation | | | | | | |
| --- | --- | --- | --- | --- | --- | --- |
| 1 | 2 | 3 | 4 | 5 | n | % |
| hydroxychloroquine |  |  |  |  | 477 | 25.17% |
| hydroxychloroquine | tocilizumab |  |  |  | 184 | 9.71% |
| hydroxychloroquine, tocilizumab |  |  |  |  | 161 | 8.50% |
| hydroxychloroquine, tocilizumab | steroid |  |  |  | 109 | 5.75% |
| azithromycin, hydroxychloroquine |  |  |  |  | 96 | 5.07% |
| hydroxychloroquine | tocilizumab | steroid |  |  | 76 | 4.01% |
| atazanavir, hydroxychloroquine |  |  |  |  | 59 | 3.11% |
| azithromycin, hydroxychloroquine, lopinavir, ritonavir |  |  |  |  | 37 | 1.95% |
| tocilizumab |  |  |  |  | 35 | 1.85% |
| atazanavir, hydroxychloroquine | tocilizumab |  |  |  | 32 | 1.69% |
| hydroxychloroquine | azithromycin |  |  |  | 31 | 1.64% |
| azithromycin | hydroxychloroquine |  |  |  | 27 | 1.42% |
| atazanavir, hydroxychloroquine, tocilizumab |  |  |  |  | 26 | 1.37% |
| azithromycin, hydroxychloroquine | tocilizumab |  |  |  | 24 | 1.27% |
| azithromycin |  |  |  |  | 24 | 1.27% |
| azithromycin, hydroxychloroquine, lopinavir, ritonavir | tocilizumab |  |  |  | 23 | 1.21% |
| atazanavir, azithromycin, hydroxychloroquine |  |  |  |  | 22 | 1.16% |
| atazanavir, hydroxychloroquine | tocilizumab | steroid |  |  | 20 | 1.06% |
| hydroxychloroquine | steroid |  |  |  | 19 | 1.00% |
| azithromycin, hydroxychloroquine, tocilizumab |  |  |  |  | 19 | 1.00% |
| steroid |  |  |  |  | 18 | 0.95% |
| tocilizumab | hydroxychloroquine |  |  |  | 11 | 0.58% |
| azithromycin | atazanavir, hydroxychloroquine |  |  |  | 10 | 0.53% |
| hydroxychloroquine, steroid |  |  |  |  | 10 | 0.53% |
| azithromycin, hydroxychloroquine, tocilizumab | steroid |  |  |  | 10 | 0.53% |
| azithromycin, hydroxychloroquine | tocilizumab | steroid |  |  | 10 | 0.53% |
| tocilizumab | steroid |  |  |  | 9 | 0.47% |
| azithromycin, hydroxychloroquine, lopinavir, ritonavir | tocilizumab | steroid |  |  | 9 | 0.47% |
| atazanavir, hydroxychloroquine, tocilizumab | steroid |  |  |  | 9 | 0.47% |
| hydroxychloroquine, steroid, tocilizumab |  |  |  |  | 8 | 0.42% |
| atazanavir, azithromycin, hydroxychloroquine | tocilizumab |  |  |  | 8 | 0.42% |
| azithromycin, hydroxychloroquine | atazanavir |  |  |  | 7 | 0.37% |
| hydroxychloroquine, lopinavir, ritonavir |  |  |  |  | 6 | 0.32% |
| tocilizumab | hydroxychloroquine | steroid |  |  | 6 | 0.32% |
| hydroxychloroquine | steroid, tocilizumab |  |  |  | 6 | 0.32% |
| hydroxychloroquine | azithromycin | tocilizumab |  |  | 5 | 0.26% |
| azithromycin | hydroxychloroquine | atazanavir |  |  | 4 | 0.21% |
| hydroxychloroquine | azithromycin, tocilizumab |  |  |  | 4 | 0.21% |
| azithromycin | hydroxychloroquine, tocilizumab | steroid |  |  | 4 | 0.21% |
| atazanavir, hydroxychloroquine | azithromycin |  |  |  | 4 | 0.21% |
| azithromycin, hydroxychloroquine | lopinavir, ritonavir |  |  |  | 4 | 0.21% |
| atazanavir, azithromycin, hydroxychloroquine | lopinavir, ritonavir |  |  |  | 4 | 0.21% |
| azithromycin, hydroxychloroquine | steroid |  |  |  | 4 | 0.21% |
| steroid | hydroxychloroquine |  |  |  | 4 | 0.21% |
| steroid | hydroxychloroquine, tocilizumab |  |  |  | 4 | 0.21% |
| azithromycin | hydroxychloroquine, tocilizumab |  |  |  | 4 | 0.21% |
| hydroxychloroquine | azithromycin | steroid |  |  | 4 | 0.21% |
| hydroxychloroquine, lopinavir, ritonavir | azithromycin |  |  |  | 4 | 0.21% |
| atazanavir, azithromycin, hydroxychloroquine | tocilizumab | steroid |  |  | ≤ 3 | ≤ 0.16% |
| steroid | atazanavir, azithromycin, hydroxychloroquine |  |  |  | ≤ 3 | ≤ 0.16% |
| hydroxychloroquine, lopinavir, ritonavir | azithromycin, tocilizumab |  |  |  | ≤ 3 | ≤ 0.16% |
| hydroxychloroquine, tocilizumab | azithromycin |  |  |  | ≤ 3 | ≤ 0.16% |
| atazanavir, azithromycin, hydroxychloroquine, tocilizumab | steroid |  |  |  | ≤ 3 | ≤ 0.16% |
| steroid | atazanavir, hydroxychloroquine |  |  |  | ≤ 3 | ≤ 0.16% |
| hydroxychloroquine | tocilizumab | azithromycin |  |  | ≤ 3 | ≤ 0.16% |
| azithromycin, hydroxychloroquine | atazanavir | tocilizumab | lopinavir, ritonavir | steroid | ≤ 3 | ≤ 0.16% |
| atazanavir, hydroxychloroquine, steroid, tocilizumab |  |  |  |  | ≤ 3 | ≤ 0.16% |
| hydroxychloroquine, lopinavir, ritonavir, tocilizumab |  |  |  |  | ≤ 3 | ≤ 0.16% |
| steroid, tocilizumab | hydroxychloroquine |  |  |  | ≤ 3 | ≤ 0.16% |
| hydroxychloroquine | azithromycin | tocilizumab | steroid |  | ≤ 3 | ≤ 0.16% |
| hydroxychloroquine | azithromycin, tocilizumab | steroid |  |  | ≤ 3 | ≤ 0.16% |
| hydroxychloroquine, steroid | tocilizumab |  |  |  | ≤ 3 | ≤ 0.16% |
| hydroxychloroquine, steroid | azithromycin |  |  |  | ≤ 3 | ≤ 0.16% |
| azithromycin | hydroxychloroquine | tocilizumab | steroid |  | ≤ 3 | ≤ 0.16% |
| atazanavir, hydroxychloroquine | azithromycin | tocilizumab | steroid |  | ≤ 3 | ≤ 0.16% |
| azithromycin, hydroxychloroquine | atazanavir | lopinavir, ritonavir |  |  | ≤ 3 | ≤ 0.16% |
| atazanavir, hydroxychloroquine, steroid | tocilizumab |  |  |  | ≤ 3 | ≤ 0.16% |
| azithromycin, hydroxychloroquine | atazanavir | lopinavir, ritonavir | tocilizumab |  | ≤ 3 | ≤ 0.16% |
| atazanavir, azithromycin, hydroxychloroquine, tocilizumab |  |  |  |  | ≤ 3 | ≤ 0.16% |
| azithromycin | hydroxychloroquine | tocilizumab | atazanavir |  | ≤ 3 | ≤ 0.16% |
| steroid, tocilizumab |  |  |  |  | ≤ 3 | ≤ 0.16% |
| azithromycin, hydroxychloroquine, tocilizumab | lopinavir, ritonavir |  |  |  | ≤ 3 | ≤ 0.16% |
| azithromycin, cobicistat, darunavir, hydroxychloroquine | lopinavir, ritonavir |  |  |  | ≤ 3 | ≤ 0.16% |
| atazanavir, hydroxychloroquine | steroid |  |  |  | ≤ 3 | ≤ 0.16% |
| hydroxychloroquine | atazanavir | tocilizumab |  |  | ≤ 3 | ≤ 0.16% |
| atazanavir, hydroxychloroquine | azithromycin | tocilizumab |  |  | ≤ 3 | ≤ 0.16% |
| atazanavir, hydroxychloroquine, tocilizumab | azithromycin |  |  |  | ≤ 3 | ≤ 0.16% |
| azithromycin, hydroxychloroquine | steroid | tocilizumab |  |  | ≤ 3 | ≤ 0.16% |
| azithromycin, hydroxychloroquine | steroid, tocilizumab |  |  |  | ≤ 3 | ≤ 0.16% |
| azithromycin, cobicistat, darunavir, hydroxychloroquine |  |  |  |  | ≤ 3 | ≤ 0.16% |
| azithromycin | atazanavir, hydroxychloroquine | tocilizumab |  |  | ≤ 3 | ≤ 0.16% |
| atazanavir, azithromycin, hydroxychloroquine | steroid |  |  |  | ≤ 3 | ≤ 0.16% |
| azithromycin, hydroxychloroquine, lopinavir, ritonavir, tocilizumab |  |  |  |  | ≤ 3 | ≤ 0.16% |
| hydroxychloroquine | tocilizumab | azithromycin | steroid |  | ≤ 3 | ≤ 0.16% |
| azithromycin | tocilizumab |  |  |  | ≤ 3 | ≤ 0.16% |
| azithromycin, hydroxychloroquine, steroid, tocilizumab |  |  |  |  | ≤ 3 | ≤ 0.16% |
| steroid | immune glob G(IgG) |  |  |  | ≤ 3 | ≤ 0.16% |
| hydroxychloroquine | lopinavir, ritonavir, tocilizumab |  |  |  | ≤ 3 | ≤ 0.16% |
| atazanavir, hydroxychloroquine | tocilizumab | azithromycin |  |  | ≤ 3 | ≤ 0.16% |
| azithromycin | hydroxychloroquine | tocilizumab |  |  | ≤ 3 | ≤ 0.16% |
| azithromycin, hydroxychloroquine, lopinavir, ritonavir, steroid |  |  |  |  | ≤ 3 | ≤ 0.16% |
| azithromycin, hydroxychloroquine, steroid | tocilizumab |  |  |  | ≤ 3 | ≤ 0.16% |
| oseltamivir | atazanavir, hydroxychloroquine | tocilizumab |  |  | ≤ 3 | ≤ 0.16% |
| hydroxychloroquine, tocilizumab | azithromycin, steroid |  |  |  | ≤ 3 | ≤ 0.16% |
| cobicistat, darunavir, hydroxychloroquine |  |  |  |  | ≤ 3 | ≤ 0.16% |
| immune glob G(IgG) |  |  |  |  | ≤ 3 | ≤ 0.16% |
| oseltamivir | atazanavir, hydroxychloroquine |  |  |  | ≤ 3 | ≤ 0.16% |
| azithromycin, hydroxychloroquine | lopinavir, ritonavir | atazanavir |  |  | ≤ 3 | ≤ 0.16% |
| azithromycin, steroid | hydroxychloroquine, tocilizumab |  |  |  | ≤ 3 | ≤ 0.16% |
| azithromycin | atazanavir, hydroxychloroquine | steroid |  |  | ≤ 3 | ≤ 0.16% |
| azithromycin, tocilizumab | hydroxychloroquine | lopinavir, ritonavir | steroid |  | ≤ 3 | ≤ 0.16% |
| tocilizumab | hydroxychloroquine | steroid | lopinavir, ritonavir |  | ≤ 3 | ≤ 0.16% |
| steroid | atazanavir, hydroxychloroquine | azithromycin | lopinavir, ritonavir |  | ≤ 3 | ≤ 0.16% |
| atazanavir, azithromycin, hydroxychloroquine, tocilizumab | lopinavir, ritonavir |  |  |  | ≤ 3 | ≤ 0.16% |
| azithromycin, hydroxychloroquine, lopinavir, ritonavir | steroid | tocilizumab |  |  | ≤ 3 | ≤ 0.16% |
| hydroxychloroquine | lopinavir, ritonavir |  |  |  | ≤ 3 | ≤ 0.16% |
| azithromycin, hydroxychloroquine, lopinavir, ritonavir | steroid, tocilizumab |  |  |  | ≤ 3 | ≤ 0.16% |
| azithromycin, hydroxychloroquine | lopinavir, ritonavir, tocilizumab |  |  |  | ≤ 3 | ≤ 0.16% |
| azithromycin | hydroxychloroquine, lopinavir, ritonavir | tocilizumab | steroid |  | ≤ 3 | ≤ 0.16% |
| azithromycin, hydroxychloroquine | atazanavir | tocilizumab | steroid |  | ≤ 3 | ≤ 0.16% |
| azithromycin, hydroxychloroquine | lopinavir, ritonavir | tocilizumab | steroid |  | ≤ 3 | ≤ 0.16% |
| hydroxychloroquine, ritonavir, tocilizumab |  |  |  |  | ≤ 3 | ≤ 0.16% |
| atazanavir, azithromycin, hydroxychloroquine | lopinavir, ritonavir, tocilizumab |  |  |  | ≤ 3 | ≤ 0.16% |
| tocilizumab | atazanavir, hydroxychloroquine | steroid |  |  | ≤ 3 | ≤ 0.16% |
| steroid | hydroxychloroquine | tocilizumab |  |  | ≤ 3 | ≤ 0.16% |
| azithromycin, hydroxychloroquine, lopinavir, ritonavir, steroid, tocilizumab |  |  |  |  | ≤ 3 | ≤ 0.16% |
| atazanavir, hydroxychloroquine | azithromycin, tocilizumab | steroid |  |  | ≤ 3 | ≤ 0.16% |
| hydroxychloroquine, tocilizumab | lopinavir, ritonavir | steroid |  |  | ≤ 3 | ≤ 0.16% |
| azithromycin | tocilizumab | steroid |  |  | ≤ 3 | ≤ 0.16% |
| hydroxychloroquine | atazanavir, tocilizumab | steroid |  |  | ≤ 3 | ≤ 0.16% |
| azithromycin, hydroxychloroquine, lopinavir, ritonavir | steroid |  |  |  | ≤ 3 | ≤ 0.16% |
| atazanavir, hydroxychloroquine | azithromycin, tocilizumab |  |  |  | ≤ 3 | ≤ 0.16% |
| steroid | atazanavir, hydroxychloroquine, tocilizumab |  |  |  | ≤ 3 | ≤ 0.16% |
| hydroxychloroquine | lopinavir, ritonavir | tocilizumab | steroid |  | ≤ 3 | ≤ 0.16% |
| steroid | hydroxychloroquine | azithromycin |  |  | ≤ 3 | ≤ 0.16% |
| hydroxychloroquine, lopinavir, ritonavir | azithromycin | tocilizumab |  |  | ≤ 3 | ≤ 0.16% |
| steroid | atazanavir, azithromycin, hydroxychloroquine, tocilizumab |  |  |  | ≤ 3 | ≤ 0.16% |
| lopinavir, ritonavir | hydroxychloroquine, tocilizumab | steroid | atazanavir |  | ≤ 3 | ≤ 0.16% |
| azithromycin | atazanavir, hydroxychloroquine, tocilizumab | steroid |  |  | ≤ 3 | ≤ 0.16% |
| atazanavir, hydroxychloroquine | tocilizumab | azithromycin, steroid |  |  | ≤ 3 | ≤ 0.16% |
| hydroxychloroquine, steroid | azithromycin | tocilizumab |  |  | ≤ 3 | ≤ 0.16% |
| azithromycin, hydroxychloroquine | atazanavir | lopinavir, ritonavir, tocilizumab | steroid |  | ≤ 3 | ≤ 0.16% |
| azithromycin, cobicistat, darunavir, hydroxychloroquine, lopinavir, ritonavir | tocilizumab | steroid |  |  | ≤ 3 | ≤ 0.16% |
| hydroxychloroquine | lopinavir, ritonavir | azithromycin, tocilizumab |  |  | ≤ 3 | ≤ 0.16% |
| azithromycin, hydroxychloroquine | tocilizumab | darunavir, ritonavir | steroid | cobicistat | ≤ 3 | ≤ 0.16% |
| tocilizumab | azithromycin | steroid |  |  | ≤ 3 | ≤ 0.16% |
| hydroxychloroquine | tocilizumab | steroid | immune glob G(IgG) |  | ≤ 3 | ≤ 0.16% |
| hydroxychloroquine | tocilizumab | steroid | azithromycin |  | ≤ 3 | ≤ 0.16% |
| hydroxychloroquine | atazanavir | azithromycin |  |  | ≤ 3 | ≤ 0.16% |
| atazanavir, hydroxychloroquine | immune glob G(IgG) |  |  |  | ≤ 3 | ≤ 0.16% |
| hydroxychloroquine, tocilizumab | azithromycin | steroid |  |  | ≤ 3 | ≤ 0.16% |
| hydroxychloroquine | lopinavir, ritonavir | steroid |  |  | ≤ 3 | ≤ 0.16% |
| atazanavir, hydroxychloroquine | tocilizumab | immune glob G(IgG) | steroid |  | ≤ 3 | ≤ 0.16% |
| azithromycin, lopinavir, ritonavir | atazanavir, hydroxychloroquine |  |  |  | ≤ 3 | ≤ 0.16% |
| azithromycin | atazanavir, hydroxychloroquine, tocilizumab |  |  |  | ≤ 3 | ≤ 0.16% |
| lopinavir, ritonavir | azithromycin, hydroxychloroquine, tocilizumab |  |  |  | ≤ 3 | ≤ 0.16% |
| azithromycin | atazanavir, hydroxychloroquine | tocilizumab | steroid |  | ≤ 3 | ≤ 0.16% |
| steroid | tocilizumab |  |  |  | ≤ 3 | ≤ 0.16% |
| azithromycin, hydroxychloroquine | cobicistat, darunavir | tocilizumab | steroid |  | ≤ 3 | ≤ 0.16% |
| steroid | azithromycin | hydroxychloroquine |  |  | ≤ 3 | ≤ 0.16% |
| darunavir, ritonavir | hydroxychloroquine |  |  |  | ≤ 3 | ≤ 0.16% |
| steroid | azithromycin |  |  |  | ≤ 3 | ≤ 0.16% |
| atazanavir, hydroxychloroquine, tocilizumab | steroid | azithromycin |  |  | ≤ 3 | ≤ 0.16% |
| hydroxychloroquine | lopinavir, ritonavir | tocilizumab |  |  | ≤ 3 | ≤ 0.16% |
| azithromycin | hydroxychloroquine | atazanavir | tocilizumab |  | ≤ 3 | ≤ 0.16% |
| azithromycin, hydroxychloroquine | immun glob G(IgG) |  |  |  | ≤ 3 | ≤ 0.16% |
| hydroxychloroquine, lopinavir, ritonavir | azithromycin | steroid |  |  | ≤ 3 | ≤ 0.16% |
| hydroxychloroquine | atazanavir, azithromycin |  |  |  | ≤ 3 | ≤ 0.16% |
| atazanavir, hydroxychloroquine | azithromycin, lopinavir, ritonavir | tocilizumab |  |  | ≤ 3 | ≤ 0.16% |
| lopinavir, ritonavir | azithromycin, hydroxychloroquine | tocilizumab |  |  | ≤ 3 | ≤ 0.16% |
| azithromycin | hydroxychloroquine | atazanavir | lopinavir, ritonavir |  | ≤ 3 | ≤ 0.16% |
| atazanavir, azithromycin, hydroxychloroquine | steroid, tocilizumab |  |  |  | ≤ 3 | ≤ 0.16% |
| hydroxychloroquine | steroid, tocilizumab | lopinavir, ritonavir |  |  | ≤ 3 | ≤ 0.16% |
| lopinavir, ritonavir | hydroxychloroquine | azithromycin |  |  | ≤ 3 | ≤ 0.16% |
| hydroxychloroquine | atazanavir, tocilizumab |  |  |  | ≤ 3 | ≤ 0.16% |
| atazanavir, hydroxychloroquine, steroid | azithromycin |  |  |  | ≤ 3 | ≤ 0.16% |
| azithromycin, steroid | hydroxychloroquine |  |  |  | ≤ 3 | ≤ 0.16% |
| cobicistat, darunavir | hydroxychloroquine |  |  |  | ≤ 3 | ≤ 0.16% |
| atazanavir, hydroxychloroquine, tocilizumab | azithromycin | steroid |  |  | ≤ 3 | ≤ 0.16% |
| hydroxychloroquine, tocilizumab | atazanavir | steroid |  |  | ≤ 3 | ≤ 0.16% |
| atazanavir, azithromycin, darunavir, hydroxychloroquine, ritonavir | tocilizumab | steroid |  |  | ≤ 3 | ≤ 0.16% |
| hydroxychloroquine | azithromycin, lopinavir, ritonavir |  |  |  | ≤ 3 | ≤ 0.16% |
| atazanavir, azithromycin, hydroxychloroquine, steroid, tocilizumab |  |  |  |  | ≤ 3 | ≤ 0.16% |
| azithromycin, tocilizumab |  |  |  |  | ≤ 3 | ≤ 0.16% |
| steroid | atazanavir, hydroxychloroquine | tocilizumab |  |  | ≤ 3 | ≤ 0.16% |
| azithromycin, hydroxychloroquine, steroid |  |  |  |  | ≤ 3 | ≤ 0.16% |
| azithromycin, hydroxychloroquine, lopinavir, ritonavir, tocilizumab | steroid |  |  |  | ≤ 3 | ≤ 0.16% |
| hydroxychloroquine | darunavir, ritonavir |  |  |  | ≤ 3 | ≤ 0.16% |
| atazanavir |  |  |  |  | ≤ 3 | ≤ 0.16% |
| hydroxychloroquine, tocilizumab | atazanavir, ritonavir | steroid |  |  | ≤ 3 | ≤ 0.16% |
| hydroxychloroquine, tocilizumab | azithromycin | lopinavir, ritonavir | steroid |  | ≤ 3 | ≤ 0.16% |
| hydroxychloroquine | azithromycin | lopinavir, ritonavir |  |  | ≤ 3 | ≤ 0.16% |
| darunavir, hydroxychloroquine, ritonavir | tocilizumab |  |  |  | ≤ 3 | ≤ 0.16% |
| azithromycin, hydroxychloroquine | lopinavir, ritonavir, tocilizumab | steroid |  |  | ≤ 3 | ≤ 0.16% |
| hydroxychloroquine | azithromycin, lopinavir, ritonavir | tocilizumab |  |  | ≤ 3 | ≤ 0.16% |
| atazanavir, tocilizumab | steroid |  |  |  | ≤ 3 | ≤ 0.16% |
| atazanavir, hydroxychloroquine, oseltamivir | tocilizumab |  |  |  | ≤ 3 | ≤ 0.16% |
| hydroxychloroquine, tocilizumab | atazanavir, steroid |  |  |  | ≤ 3 | ≤ 0.16% |
